# Characterizing the detectable and invisible fractions of genomic loci under balancing selection

**DOI:** 10.1093/molbev/msag228

**Published:** 2026-10-05

**Authors:** Débora Y C Brandt, Aida M Andrés, Tim Connallon, Max Reuter

**Affiliations:** Research Department of Genetics, Evolution and Environment, University College London, London WC1E 6BT, UK; UCL Genetics Institute, University College London, London WC1E 6BT, UK; Research Department of Genetics, Evolution and Environment, University College London, London WC1E 6BT, UK; UCL Genetics Institute, University College London, London WC1E 6BT, UK; School of Biological Sciences, Monash University, Clayton, Victoria 3800, Australia; Research Department of Genetics, Evolution and Environment, University College London, London WC1E 6BT, UK; Centre for Life′s Origins and Evolution, University College London, London WC1E 6BT, UK

**Keywords:** genetic variation, overdominance, antagonistic pleiotropy, sexual antagonism, theory, simulations

## Abstract

Balancing selection refers to scenarios where selection maintains genetic polymorphisms affecting fitness and its components. Empirical studies have documented specific cases of balancing selection, yet the general prevalence of balanced polymorphisms within genomes remains a topic of longstanding debate. Although genome-wide scans for signals of balancing selection suggest that it is rare, current methods are notoriously conservative and may identify only a small and unrepresentative fraction of loci evolving under balancing selection. What, then, are the proportions of loci under balancing selection that are detectable versus invisible to genome scans? Here, we address this question using a combination of analytical modeling and population genetic simulations. Our results provide quantitative support for the intuition that a large fraction of loci under balancing selection will be invisible to standard tests for balancing selection, with the detectable loci representing a biased subset with large and symmetrical fitness effects on different fitness components. Quantifying power across parameters that vary between species also shows that the detectable and invisible fractions of genomic loci under balancing selection are likely to differ substantially between organisms. For example, a *Drosophila melanogaster*-like ratio of mutation to recombination rates (∼0.1) reduces detection power by roughly a quarter of the power expected with a human-like ratio (∼1). This, combined with other evidence suggesting that balancing selection could be common in *D. melanogaster*, showcases how our appreciation of the prevalence of balancing selection is limited by our ability to detect its genomic signals, especially in those species where it might be common.

## Introduction

Balancing selection occurs when natural selection actively favors the maintenance of genetic polymorphism. This can arise from several specific contexts of selection, including intrinsic fitness advantages of being heterozygous (ie “heterozygote advantage” or fitness “overdominance” (Fisher 1922)), genetic tradeoffs between different fitness contexts or environments (eg “antagonistic pleiotropy” between fitness components (Curtsinger et al. 1994); “sexually antagonistic” tradeoffs between sexes (Kidwell et al. 1977)), tradeoffs between temporal or spatially varying environments (Levene 1953; Haldane and Jayakar 1963), or negative-frequency-dependent selection, where genotypes become more favorable when rare (Wright 1942; Fitzpatrick et al. 2007).

Loci under balancing selection are expected to be more polymorphic than loci evolving neutrally or under directional selection (though exceptions can occur when selection is weak or allele frequency equilibria under balancing selection are close to zero or one (Robertson 1962; Williamson et al. 2004)). When equilibrium frequencies are intermediate (eg very roughly between 0.2 and 0.8 (Hedrick 1999)) and balancing selection is strong relative to genetic drift, the balanced polymorphisms should persist far longer than neutrally evolving polymorphisms (Nei and Roychoudhury 1973), potentially over timescales spanning speciation events (Klein et al. 1998). Whatever their age, balanced polymorphisms should contribute disproportionately to standing genetic variation for fitness or fitness components (eg for survival, fertility, or longevity), with even modest numbers of such loci contributing substantially to fitness variation within populations (Crow 1952, 1987; Charlesworth 2015; Zajitschek and Connallon 2018; Connallon and Czuppon 2026).

The importance of balancing selection has, nevertheless, been a subject of longstanding debate in evolutionary biology. As an early and vociferous proponent, Dobzhansky (1955) argued that selection should often preserve genetic variation due to the greater versatility of heterozygous, relative to homozygous individuals in perpetually changing environments. Yet, by the close of the 20th century, high levels of molecular genetic diversity were reported by studies based first on allozymes (Lewontin and Hubby 1966; Lewontin 1974) and subsequently DNA sequences (Kreitman 1983). The pervasiveness of these polymorphisms made Dobzhansky's “balance hypothesis” an implausible general explanation for molecular genomic diversity. Instead, the emerging neutral theory (Kimura 1968, 1983; King and Jukes 1969) provided a simpler and more powerful explanation for the ubiquity of molecular genetic diversity, effectively sidelining balancing selection as irrelevant for all but a few exceptional loci within the genome (see p. 199 of Lewontin (1974); Hedrick (2012)).

More recent developments in evolutionary genetics have, however, prompted a resurgence in enthusiasm for balancing selection. First, the coupling of field studies with genomics has identified exceptionally clear cases of balancing selection that maintains conspicuous phenotypic polymorphisms. Beyond classic examples like sickle cell anemia (Allison 1954), modern case studies include a pleiotropic tradeoff between viability and mating success that maintains horn size polymorphism in Soay sheep (mapped to the *RXFP2* gene; Johnston et al. (2013)), a sexually antagonistic polymorphism in cuticular hydrocarbons of *Drosophila serrata* (in the *DsFAR2-B* gene; Rusuwa et al. (2022)), alleles affecting age of maturation in Atlantic salmon (the *VGLL3* gene; Barson et al. (2015)), temporally varying selection that maintains genetic polymorphism for insecticide resistance in *Drosophila melanogaster* (Karageorgi et al. 2025), and many others (reviewed in Bitarello et al. (2023); Ruzicka et al. (2025)). Second, the advent of high-throughput sequencing techniques and sophisticated analytical methods for detecting population genetic signatures of balancing selection has provided new means of identifying candidate loci under long-term balancing selection (reviewed in Fijarczyk and Babik 2015; Bitarello et al. 2023). Applied to the human genome, such methods have provided strong evidence for long-term balancing selection in both African and European populations (Bitarello et al. 2018). Third, detailed lab studies of natural genetic variation in life-history traits suggest that *Drosophila* populations harbor higher levels of genetic variation than can be explained by models of mutation-selection balance (Charlesworth and Hughes 2000; Charlesworth 2015; Sharp and Agrawal 2018). Balancing selection is a prime candidate to account for this apparent excess of genetic variation in life-history traits. If the same is true in other species, balancing selection may maintain substantial amounts of genetic and trait variation in natural populations.

While recent genome scans have identified putative balanced polymorphisms, it remains unclear how accurately they capture the true extent of balancing selection. The typically small fraction of loci with statistically significant signals of balancing selection (eg <1% of the genome in humans (Bitarello et al. 2018)) might reflect either the rarity of balancing selection across the genome or methodological limitations to detecting it. Most notably, classical methods cannot detect balancing selection unless intermediate-frequency polymorphisms are maintained over exceptionally long timescales. Loci segregating for evolutionarily “young” balanced polymorphisms cannot easily be identified through population genomic approaches alone (but see Isildak et al. (2021) for a potentially promising approach), and old polymorphisms can likewise be missed due to limited data quality or other attributes that vary among species (eg mutation and recombination rates; effective population sizes) and may influence the strength of balancing selection signals. The balanced polymorphisms that are detected are, therefore, likely to represent only a minor, potentially unrepresentative, subset of loci evolving under balancing selection. In fact, a recent genome-wide analysis suggests there are hundreds of nonsynonymous polymorphisms shared among human populations due to recent balancing selection, even though the method can detect only an aggregate signal across many loci rather than identify individual targets of balancing selection (Soni et al. 2022). While it remains an open question what proportion of these loci ultimately results in long-term stable balanced polymorphisms that are individually identifiable, this analysis suggests that at least recent episodes of balancing selection are widespread in the human genome.

Here, we aim to put the debate over the prevalence of balancing selection on a sounder conceptual and quantitative footing by evaluating factors affecting opportunities for long-term balancing selection and its detectability. It is obviously impossible to make general predictions about the proportion of a genome that is a potential target of balancing selection. Yet it is possible to assess—under a given model of balancing selection—how the population genetic parameters of genetic drift and selection will affect: (i) the long-term maintenance of polymorphisms, and (ii) the proportion of loci that develop signatures of balancing selection that are clear enough to be detectable using contemporary population genomic methods.

We address these questions while considering 3 mechanisms of balancing selection: overdominance, antagonistic pleiotropy, and sexually antagonistic selection, each of which has been empirically documented in natural populations (eg Allison 1954; Johnston et al. 2013; Rusuwa et al. 2022). These mechanisms represent a subset of balancing selection mechanisms (eg Prout 2000; Glémin 2021; Ruzicka et al. 2025), yet their evolutionary dynamics are representative of a broader array of balancing selection scenarios, which allows our predictions to be generalized. We begin by briefly reviewing predictions from population genetics theory, highlighting constraints associated with maintaining long-term stable polymorphisms under each mechanism of balancing selection. We then present simulations under each mechanism that explore the genomic signals associated with long-term balanced polymorphisms and assess how these signals translate into statistical power to infer the presence of balancing selection at these loci. Our analyses reveal how the maintenance of balanced polymorphisms and their detectability depend on the properties of the genetic polymorphism itself, as well as the genetic and demographic features of the species. They also provide a quantitative appreciation for the extent to which loci subject to balancing selection are detectable and suggest the biases in the specific types of balanced polymorphisms that are most and least likely to be identified.

## Results

### Long-term maintenance of balanced polymorphisms: theoretical considerations

We start by reviewing theoretical predictions regarding the conditions under which genetic polymorphisms are stably maintained due to overdominant selection (OD), antagonistic pleiotropy (AP), or sexually antagonistic selection (SA). Our models focus on bi-allelic, diploid loci (Table 1), and we assume that individuals mate at random and selection at each locus remains constant over time. Violations of these assumptions tend to restrict conditions for balancing selection (reviewed in Ruzicka et al. (2025), but see Wittmann et al. (2023) for signals of long-term balancing selection under temporally fluctuating selection). Full details of the models are presented in Appendix S1.

**Table 1:** Fitness per genotype for each model of selection.

	Genotype		
	*A*_1_ *A*_1_	*A*_1_ *A*_2_	*A*_2_ *A*_2_
Overdominance	1 – *s*_1_	1	1 – *s*_2_
Antagonistic pleiotropy			
Fitness component 1	1	1 – *s*_2_*h*	1 – *s*_2_
Fitness component 2	1 – *s*_1_	1 – *s*_1_*h*	1
Overall fitness	1 – *s*_1_	(1 – *s*_1_*h*)(1 – *s*_2_*h*)	1 – *s*_2_
Sexual antagonism			
Female fitness	1	1 – *s*_2_*h*	1 – *s*_2_
Male fitness	1 – *s*_1_	1 – *s*_1_*h*	1

Long-term maintenance of a polymorphism under these models of selection requires 2 conditions. First, the combination of allelic fitness effects must actually generate balancing selection that persists over time. Second, the resulting balancing selection must be stronger than genetic drift for the polymorphism to be stably maintained over time. We deal with each condition in turn.

As far as the general criteria for balancing selection are concerned, OD is the most permissive scenario. In outbred populations, heterozygotes having higher fitness than both homozygotes (OD) invariably leads to balancing selection (Fisher 1922). In contrast, AP and SA lead to balancing selection under only a subset of parameter conditions (Fig. 1). AP and SA each involve tradeoffs between the fitness effects of alleles in different contexts of selection (between fitness components and between sexes, respectively). Considering a locus with alleles *A*_1_ and *A*_2_, *s*_1_ represents the homozygous cost associated with the *A*_1_ allele, *s*_2_ is the homozygous cost associated with the *A*_2_ allele, and dominance (*h*) defines the degree to which these costs manifest in heterozygotes (Fig. 1a, Table 1). For simplicity, we focus on cases in which the dominance coefficient ranges from *h* = 0.5, implying co-dominance of the alleles, to *h* = 0, implying complete recessivity of these costs (for generalizations beyond these conditions, see Kidwell et al. (1977); Curtsinger et al. (1994)). Note that dominance values within the range 0 < *h* < 0.5 correspond to “dominance reversals” (Connallon and Chenoweth 2019; Reid 2022; Grieshop et al. 2024), where recessivity or dominance is not a fixed property of one allele but each allele is (partially) recessive in contexts where it is harmful and (partially) dominant in contexts where it is favorable.

**Figure 1:**
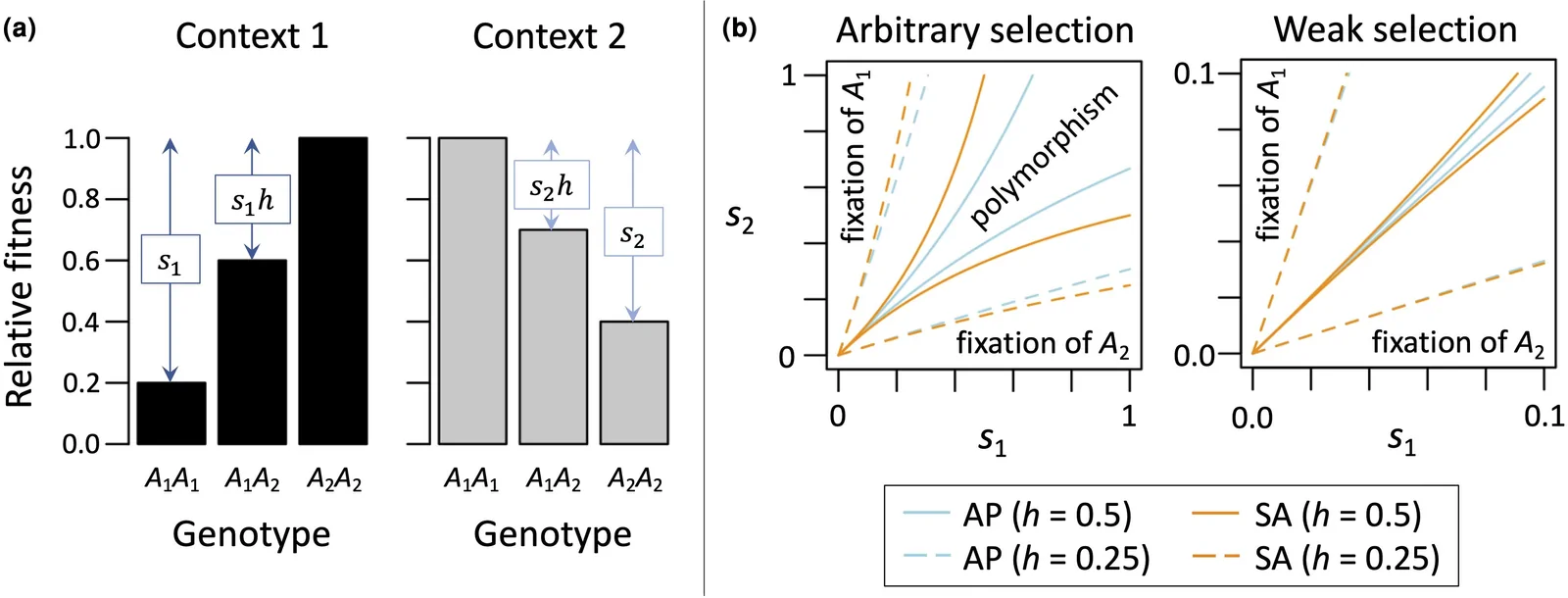
Conditions for balancing selection under antagonistic pleiotropy (AP) and sexually antagonistic selection (SA). Panel a) Visual summary of the AP and SA model parameters, with the example showing *s_1_* = 0.8, *s_2_* = 0.6, and *h* = 0.5. The 2 bar charts show the relative fitness of each of the 3 genotypes of a locus across 2 contexts of selection (ie different fitness components or sexes), where the *A*_1_ allele is selectively disfavored in one context and the *A*_2_ allele is disfavored in the other. Panel b) Criteria for balancing selection as a function of selection (*s*_1_ and *s*_2_) and dominance (*h*) parameters. The regions between each pair of curves (based on color and line type) show conditions where selection favors the maintenance of polymorphism (ie where there is balancing selection). Predictions are shown when there is no dominance between alleles (ie alleles are co-dominant: *h* = 0.5), and when there is a beneficial reversal of dominance (*h* = 0.25), where negative allelic effects are partially recessive to beneficial effects. The left panel shows the most general case with *s*_1_ and *s*_2_ ranging from 0 to 1; the right panel zooms into the bottom-left region, where selection coefficients are weak (0 to 0.1). Panel b) is modified from Connallon and Chenoweth (2019), with curves based on standard modeling results (see Kidwell et al. 1977; Curtsinger et al. 1994).

Balancing selection arises consistently in the SA and AP scenarios when the homozygous costs of each allele are symmetric in magnitude (*s*_1_ is roughly equal to *s*_2_), and the criteria for balancing selection expand with both the strength of selection and the strength of the dominance reversal (ie with *s*_1_ and *s*_2_ increasing and *h* decreasing; Fig. 1b). When selection is weak and there is no dominance reversal, only a small proportion of the parameter space will generate balancing selection. In this case, the proportion of random parameter combinations that leads to balancing selection is roughly $f_{bal}\approx \frac{2}{3}\bar{s}$ under AP and $f_{bal}\approx \frac{4}{3}\bar{s}$ under SA, where $\bar{s}$ represents the average fitness effect ($\bar{s}=\frac{s_{max}}{2}$ for parameters drawn uniformly from the parameter space, with $s_{max}$ representing the maximum selection coefficient $0\leq s_{1},s_{2}\leq s_{\max}$; $s_{\max}\ll 1$ when selection is consistently weak). With dominance reversals (*h* < 0.5) and weak selection, the proportions expand to approximately $f_{bal}\approx \frac{1-2h}{1-h}$ for both AP and SA, which corresponds to $f_{bal}\approx \frac{2}{3}$ when *h* = 0.25 (as in Fig. 1b) and $f_{bal}=1$ for a complete dominance reversal (*h* = 0) (Eq. A9 and A10 in Appendix S1).

The second condition for long-term stability of a balanced polymorphism is that selection toward a polymorphic equilibrium (which we denote as $\hat{\;p}$) must be strong enough to prevent fixation of one or the other allele by genetic drift. The required strength of selection depends on the effective size of the population ($N_{e}$) and the net strength of selection to the equilibrium (*α*), which differs between the mechanisms of balancing selection ($\alpha =s_{1}+s_{2}$ under the OD model, where *s*_1_ and *s*_2_ represent the fitness reductions of *A*_1_ and *A*_2_ homozygotes relative to the heterozygotes; $\alpha =(s_{1}+s_{2})(1-2h)+2s_{1}s_{2}h^{2}$ and $\alpha =\frac{1}{2}(s_{1}+s_{2})(1-2h)+2s_{1}s_{2}h^{2}$ under the AP and SA models, respectively, with the SA and AP parameters, $s_{1}$, $s_{2}$, and *h*, as depicted in Fig. 1a; see Appendix S2 for derivations of the equations for *α*).

The stationary distribution of the allele frequencies for a locus under balancing selection and drift (ie the predicted long-term probability distribution of allele frequency states) provides an intuitive measure of balancing selection's ability to maintain polymorphism in the presence of genetic drift (Robertson 1962; Rice 2004). Balancing selection reliably maintains polymorphism when the allele frequency probability densities are much greater near the deterministic equilibrium ($\hat{\;p}$) than near the boundaries (ie close to zero and one). Heuristically, balancing selection can be regarded as effectively strong in cases where the probability density around $\hat{\;p}$ is at least ten times greater than the density near the boundaries (Appendix S2). Under this definition, balancing selection is effectively strong when $\alpha >\alpha_{\min}$, where:


(1) $$\begin{array}{c} \alpha_{\min}=\{\begin{array}{cc} \frac{\log\;(20N_{e}\hat{\;p})}{2N_{e}{\hat{\;p}}^{2}} & \mathrm{for}\;\hat{\;p}\leq 0.5\\ \frac{\log\;(20N_{e}(1-\hat{\;p}))}{2N_{e}{\left(1-\hat{\;p}\right)}^{2}} & \mathrm{for}\;\hat{\;p}\geq 0.5 \end{array} \end{array}$$

Figure 2a illustrates how the effective population size and equilibrium allele frequencies under balancing selection ($N_{e}$ and $\hat{\;p}$) interact to determine criteria for effectively strong balancing selection (where $\alpha >\alpha_{\min}$). As can be seen in the figure, these conditions are most permissive in large populations with intermediate equilibria (note that $\alpha_{\min}$ is always minimized at $\hat{\;p}=0.5$).

**Figure 2:**
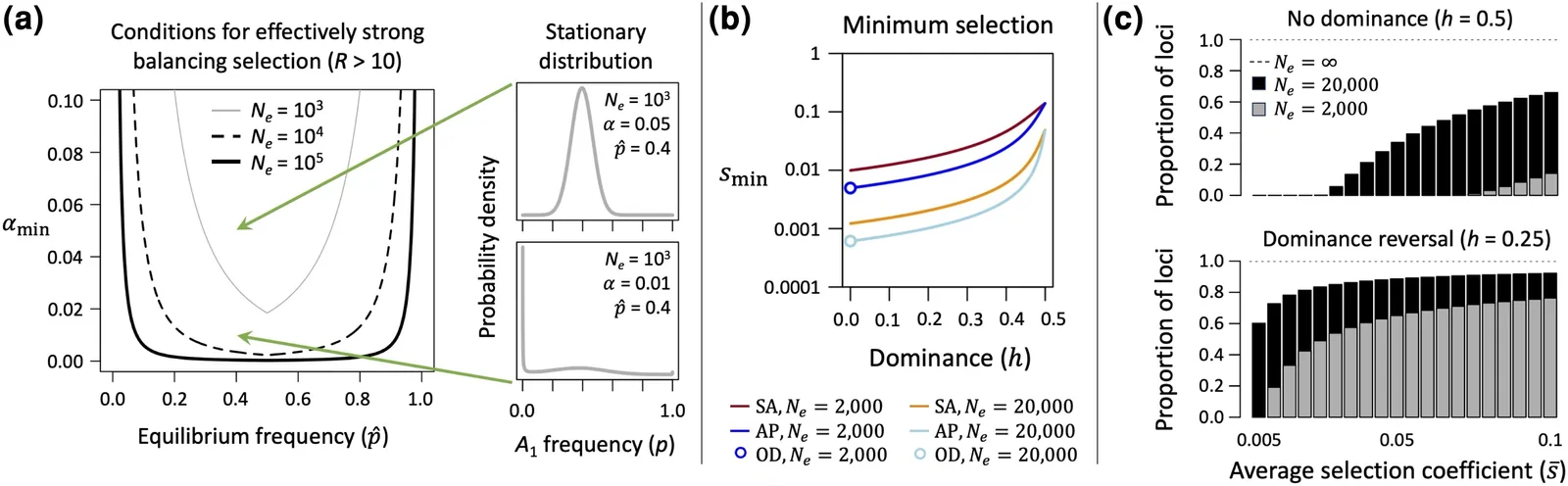
Effectively strong balancing selection. Panel a) Illustrates the thresholds for effectively strong balancing selection relative to genetic drift ($\alpha_{\min}$; see Equation (1) and the surrounding text) for 3 population sizes (*N_e_*) and a spectrum of allele frequency equilibrium conditions under balancing selection ($0<\hat{\;p}<1$). Regions above each curve denote combinations of *α* and $\hat{\;p}$ that generate effectively strong balancing selection (ie where the probability density for the stationary distribution is at least a 10-fold higher near the polymorphic equilibrium, $\hat{\;p}$, than near the allele frequency boundaries of zero and one, which correspond to fixation). Two special cases of the stationary distribution are shown: one where selection is ineffective ($\alpha <\alpha_{\min}$, and density is concentrated near *zero*) and one where selection is effective ($\alpha >\alpha_{\min}$, and density is concentrated near the equilibrium, $\hat{\;p}=0.4$). b) shows the minimum selection coefficients for each model that allow for effective balancing selection (the minimum is defined at $\hat{\;p}=0.5$, where conditions for effective balancing selection are most permissive). c) The proportion of loci under AP balancing selection that meet the criteria for effectively strong balancing selection. Results are based on randomly drawing 10^7^ sets of selection coefficients (*s*_1_ and *s*_2_) from independent uniform distributions between 0 and $s_{\max}$, where $\bar{s}=\frac{1}{2}s_{\max}$ is the average selection coefficient. Bar heights were calculated as the number of parameter sets that met the criterion for effectively strong balancing selection ($0<\hat{\;p}<1$ and $\alpha >\alpha_{\min}$), divided by the number of parameter sets that generated balancing selection ($0<\hat{\;p}<1$).

Because *α* is defined differently for each model of selection (see above), thresholds for effectively strong balancing selection correspond to different absolute strengths of selection under the AP, SA, and OD scenarios. For example, the minimum values of *s*_1_ and *s*_2_ that are required for $\alpha >\alpha_{\min}$ (shown in Fig. 2b) are largest for the SA model, smallest for the OD model, and intermediate for the AP model (SA and AP cases are equal when *h* = 0.5, and AP and OD cases are equal when *h* = 0; see Fig. 2b).

A reduction of *N_e_* amplifies drift and restricts the parameter space of effectively strong balancing selection (Fig. 2c), particularly so when dominance reversals are weak or absent. For example, when *h* = 0.5 and *N_e_* = 2,000, SA and AP alleles cannot be reliably maintained polymorphic even by moderately strong selection (eg *s*_1_ ∼ *s*_2_ ∼ 0.05, corresponding to a 5% difference between the homozygote genotypes in each fitness component). Larger *N_e_* and/or dominance reversals (*h* < 0.5) make long-term maintenance of polymorphism much more likely (Fig. 2c). Population genetic simulations (see Methods for simulation details) confirm these general predictions (Figures S2, S4, S6). They further illustrate how the notion of maintaining long-term stable polymorphisms depends on the timescale of the observation: the range of parameter values that maintains polymorphism for 16*N_e_* generations is significantly narrower than the range that maintains polymorphism for 8*N_e_* generations (Figure S2c,d).

### Empirical signatures of long-term balancing selection

The theory presented above summarizes factors that promote or constrain the maintenance of polymorphism at individual sites subject to balancing selection. Assessing the detectability of balancing selection requires additional considerations, because genomic scans for balancing selection do not focus on the balanced site itself but on signals of elevated genetic diversity at sites physically linked to the selected site. These signatures build up in the presence of long-term balancing selection, where the balanced polymorphism segregates for much longer than expected under neutrality (ie the time to the most recent common ancestor, TMRCA, at the selected site is much greater than the neutral expectation of 4*N_e_* generations). Genomic regions with an old TMRCA owing to balancing selection are expected to deviate from the genomic background in 2 ways. First, they are expected to show an elevated density of linked neutral polymorphisms (and a concomitant deficit of substitutions relative to closely related outgroups) due to the long timespans over which mutations can accumulate since the TMRCA in the region and their lower probability of going to fixation due to linkage with the balanced site (DeGiorgio et al. 2014). Second, they are expected to show an excess of intermediate-frequency alleles when compared with the neutral expectation for unlinked sites, as linked neutral variants tend to segregate at frequencies similar to those of the balanced polymorphism (see eg Fig. 1 in Bitarello et al. (2018)). These properties will be reflected in the local site-frequency spectrum (SFS) and provide the signal exploited by current population genomic methods for detecting long-term balancing selection (eg Bitarello et al. 2018; Cheng and DeGiorgio 2020).

In the following section, we will explore how the presence and intensity of the signal, and thus our statistical power to identify regions under balancing selection, depends on the properties of the balanced polymorphism itself (ie the nature and strength of balancing selection, dominance, and the resulting equilibrium frequency of the balanced polymorphism), and factors that affect population genomic data, including the effective population size, and mutation and recombination rates, which vary among species and genomic regions.

We can get a preliminary sense of some of the key factors affecting neutral diversity near a long-term balanced polymorphism by quantifying the expected spike in heterozygosity near a balanced site under strong selection, which is analytically tractable. Consider a balanced polymorphism with alleles *A*_1_ and *A*_2_ maintained stably at deterministic equilibrium frequencies, $\hat{\;p}$ and $1-\hat{\;p}$, respectively. Building on previous work (see p. 103 in Rice (2004)), one can derive the expected width of the sequence surrounding the balanced site in which neutral polymorphisms will have an expected level of heterozygosity that is at least *K* times greater than the expected heterozygosity at unlinked neutral sites elsewhere in the genome (4*N_e_µ*, where *µ* is the mutation rate per nucleotide site). The width of this region, in kilobases (kb), is:


(2) $$\begin{array}{c} L_{K}\approx \frac{2\hat{\;p}(1-\hat{\;p})}{10^{3}(K-1)N_{e}r} \end{array}$$

where *r* represents the recombination rate per nucleotide site (see Appendix S3). Inspection of Equation (2) shows that the width of elevated diversity surrounding the balanced polymorphism increases with the equilibrium heterozygosity of the selected site (with $2\hat{\;p}(1-\hat{\;p})$), which is maximized when balanced alleles are maintained at equal frequencies ($\hat{\;p}=0.5$), and it decreases with the population-scaled recombination rate per nucleotide site ($N_{e}r$). Thus, we expect empirical signals of long-term balancing selection to be strongest when $\hat{\;p}$ is intermediate and $N_{e}r$ is small, owing to small $N_{e}$ and/or small *r*.

### Power to detect balancing selection in simulated data

While the theoretical predictions above lend intuition, they ignore the reality that allele frequencies at the selected site fluctuate over time (eg due to drift) and overlook some of the empirically distinctive features of the SFS that are used by statistical methods for detecting balancing selection in genomic scans, including the actual number of segregating linked variants and the shape of the SFS around a balanced site relative to the genome as a whole. Allele frequency fluctuations at the balanced polymorphic site are expected to reduce linked diversity relative to the more idealized case (as in Equation (2)) where the alleles are maintained at invariant equilibrium frequencies (for allele frequency fluctuations caused by temporally varying selection, see Wittmann et al. (2023)). Drift-mediated allele frequency fluctuations at the selected site should be minimal provided $N_{e}\alpha$ is large and the equilibrium ($\hat{\;p}$) is intermediate, while fluctuations increase as $N_{e}\alpha$ and $\hat{\;p}(1-\hat{\;p})$ decline (Charlesworth and Charlesworth 2010, pp. 354 to 355; Connallon and Czuppon 2026).

To provide an assessment of statistical power for detecting balancing selection candidates, we turned to population genetic simulations. We ran extensive simulations (see Methods for details) with population-scaled values of *N_e_µ* and *N_e_r* chosen to roughly match those of humans and fruit flies—intensively studied species that may be representative of vertebrates and insects. We note that these simulations are not intended to provide specific power estimates for these species, since our models do not match their demographic histories (eg the long-term *N_e_* of human populations is smaller than 20,000 and their detailed demographic history generally increases power compared with the stable effective population size simulated here; see Discussion). Rather, they are presented as toy examples that allow us to evaluate effects of relevant genomic parameters that vary among species. We first assessed power for a human-like “reference” set of parameters (*N_e_* = 20,000, *µ* = *r* = 10^−8^, age of polymorphism *T* = 8*N_e_* = 160,000 generations), which we will later use as a point of comparison with simulations using different population parameters. We assessed the power of the NCD method (Bitarello et al. 2018) to identify balancing selection in population genetic simulations of 10Kb chromosomes with a centrally located site under balancing selection and neutral variation in the flanking regions (see Methods). The results obtained for NCD should be representative of those obtained with other contemporary methods for detecting long-term balancing selection, which use similar approaches; additional results using the BalLeRMix method (Cheng and DeGiorgio 2020) are presented in the Supplementary Results and Figure S13. We provide these to add some generality beyond NCD. The information is not meant (nor should it be used) to assess the relative power or the 2 methods for particular applications; thorough power analyses should be performed for this purpose.

Like other methods, NCD tests for an enrichment of polymorphism in the SFS by calculating, across all SNPs in a window, the average deviation of allele frequency from an intermediate “target frequency”. We used a target frequency 0.5 for NCD, mimicking a real-world application of the approach where the real equilibrium frequency is unknown (Bitarello et al. 2018). For each set of parameters, we generated 100 replicate simulations in which the balanced polymorphism segregated until the end of the simulation (8*N_e_* or 16*N_e_* generations, thus conditioning on the maintenance of polymorphism; for results on the probability of maintenance over these time durations, see Figures S1–S6). From these, we estimated power as the proportion of simulations that resulted in a statistically significant signal of balancing selection at *P* < 0.01, measured by NCD.

We evaluated the effects of our simulation parameters on power through multiple logistic regressions (Generalized Linear Models (GLMs) with binomial error) with counts of successful and unsuccessful detections among the 100 trials of our simulations as dependent variables. The GLMs model power on the log-odds scale and allow us to estimate and test the effects of the simulation parameters while taking into account the fact that power is bounded between 0 and 1, as well as the binomial error of successful/unsuccessful detection of balancing selection (see Methods for more details).

In our simulations, the power to detect balancing selection is relatively low overall for our set of parameters, and this is true regardless of the exact method used for detection (Figure S13). However, power varied considerably across parameter conditions in ways that broadly align with theoretical expectations. Among our human-like reference simulations, the variable that most strongly predicts power is the equilibrium frequency of the balanced polymorphism $\hat{p}$, with the likelihood of detecting balancing selection increasing as the equilibrium approaches an intermediate frequency (ie $\hat{\;p}\to 0.5$; Fig. 3a). Across these reference simulations, the average power is 0.23 at $\hat{\;p}=0.5$, while power drops to 0.01 with $\hat{\;p}=0.07$. Fitting a binomial GLM with equilibrium frequency ($\hat{\;p}$), selection strength (*α*), selection model, and their interactions as predictors of detection power, we found that the equilibrium frequency explains more than 75% of the variance in power across our simulations (Table 2). More specifically, the odds of successfully detecting balancing selection are estimated to increase by more than 80% with every 10% increase in the equilibrium frequency of the minor allele at the selected site, up to $\hat{\;p}$ = 0.5 (*b*_eq. freq._ = 6.355 on log-odds scale, estimated for OD). These results may seem trivial considering we ran NCD using a target frequency of 0.5, but in fact the equilibrium frequency remains the strongest predictor of power even when we ran NCD with the true equilibrium frequency as the target frequency, or when we ran BalLeRMix allowing it to optimize the equilibrium frequency (but not when BalLeRMix is provided with the true equilibrium frequency; see Supplementary Results).

**Figure 3:**
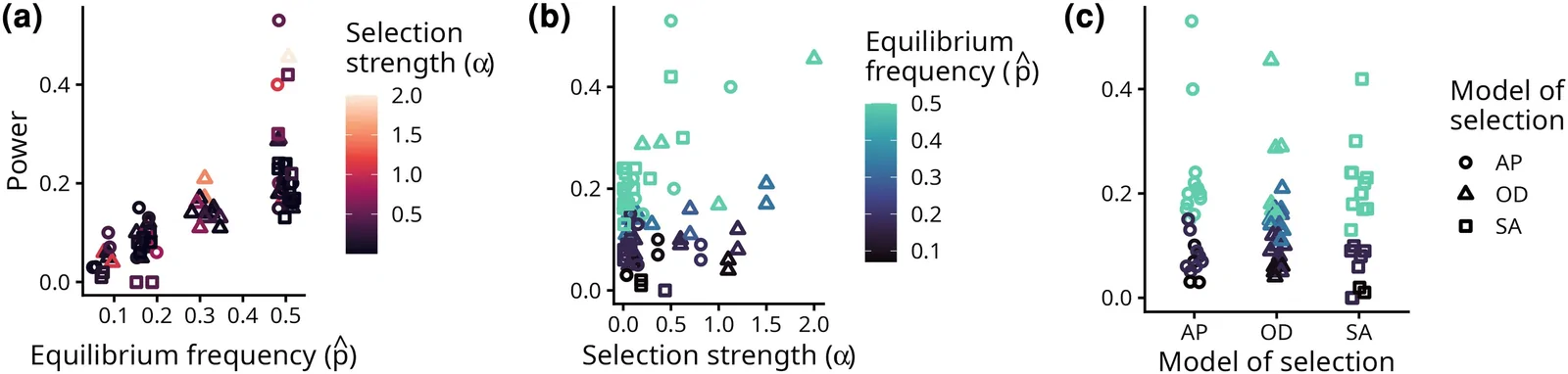
Power as a function of equilibrium frequency ($\hat{\;p}$, a), selection strength (*α*, b), and model of selection (c) for the reference simulations. On a and c points were horizontally jittered for visualization purposes. The 3 panels show the same data points, with power on the vertical axis plotted against different horizontal axes of variation.

**Table 2:** Binomial GLM of the number of instances of successful/unsuccessful detections of balancing selection in the reference set of simulations, as a function of equilibrium frequency ($\hat{\;p}$), selection strength (*α*), selection model, and their interactions.

Predictor	Deviance	% deviance explained	*P*-value
$\hat{\;p}$	781.59	75.9%	<0.001
*α*	66.37	6.4%	<0.001
$\hat{\;p}$ * selection model	14.15	1.4%	<0.001
*α* * selection model	19.58	1.9%	<0.001

Only terms with *P*-value <0.05 and proportion of deviance explained > 0.01 are shown. For a complete table and model coefficients, see Supplementary Results.

Power also increases with the strength of selection (Fig. 4), but this effect depends on interactions with other parameters. The positive effect of selection strength on power is observed for all selection models at intermediate equilibrium frequencies but declines under AP and OD, when equilibrium frequencies of the minor allele at the selected site are lower (Fig. 3b and 4b). Beyond these interactive effects, however, the model of selection does not impact the overall power of detecting balancing selection with NCD (Table 2, Fig. 3c, but see Table S14 and Table S20 in Supplementary Results for a weak effect of model of selection on BalLeRMix power).

**Figure 4:**
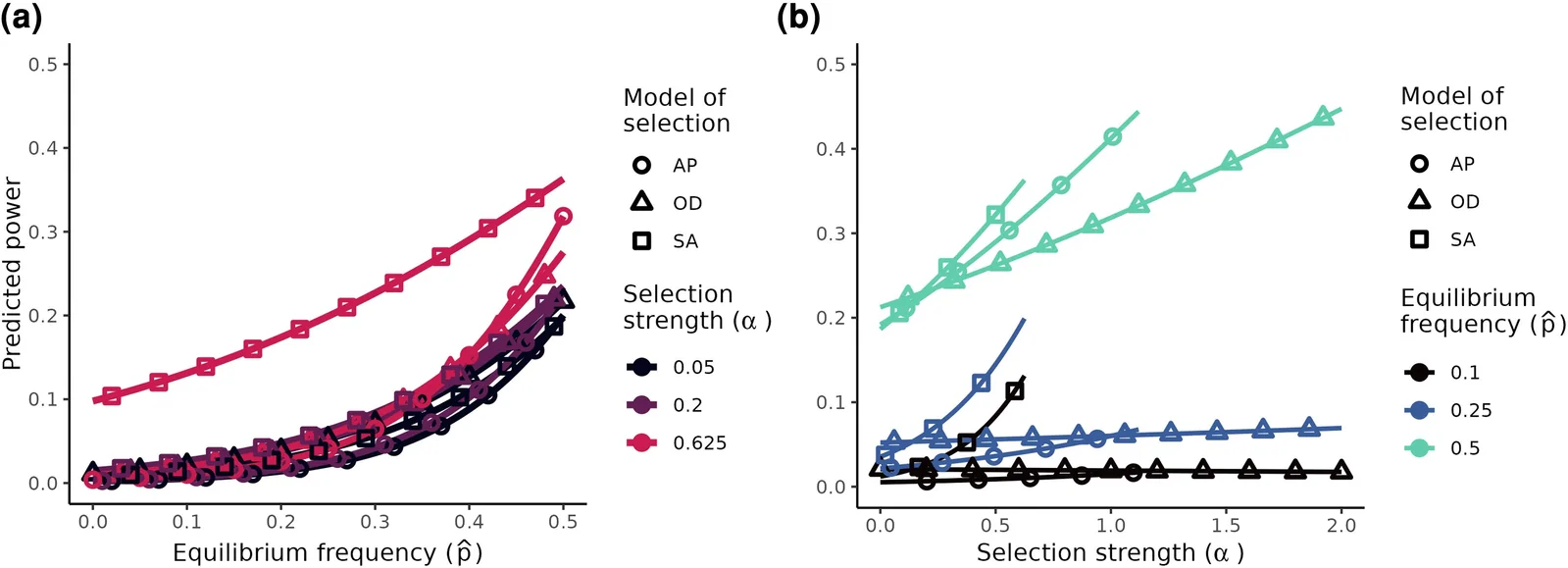
Power predicted by the generalized linear model fitted to the data for standard simulations as a function of equilibrium frequency ($\hat{\;p}$, a) and selection strength (*α*, b). Results are shown for different parameter combinations as indicated by the right-hand legend of each panel. Maximum strength of selection shown for each model of selection is the maximum strength of selection included in our simulated data.

As current methods for inferring targets of long-term balancing selection rely largely on a signal of excess polymorphism and intermediate frequencies in the SFS, power should increase in conditions that generate a larger and wider signature of excess polymorphism and decrease under those that restrict the signal. To explore these effects, we ran additional simulations under OD while varying the recombination and mutation rates, the effective population size, and the age of the balanced polymorphism.

As expected, simulations show that power consistently decreases at higher recombination rates (Fig. 5a; Table 3; Tables S3, S9, S15 and S21). More specifically, the model predicts that a 10-fold increase in recombination rate decreases the odds of detection by roughly 70% (Table 3, b values). Conversely, power increases slightly but significantly with a higher mutation rate per nucleotide site (Fig. 5b, Tables S4, S10, S16, S22), with the odds of successfully detecting balancing selection growing by about 1.6% for each 10-fold increase in mutation rate (Table 3, *b* value).

**Figure 5:**
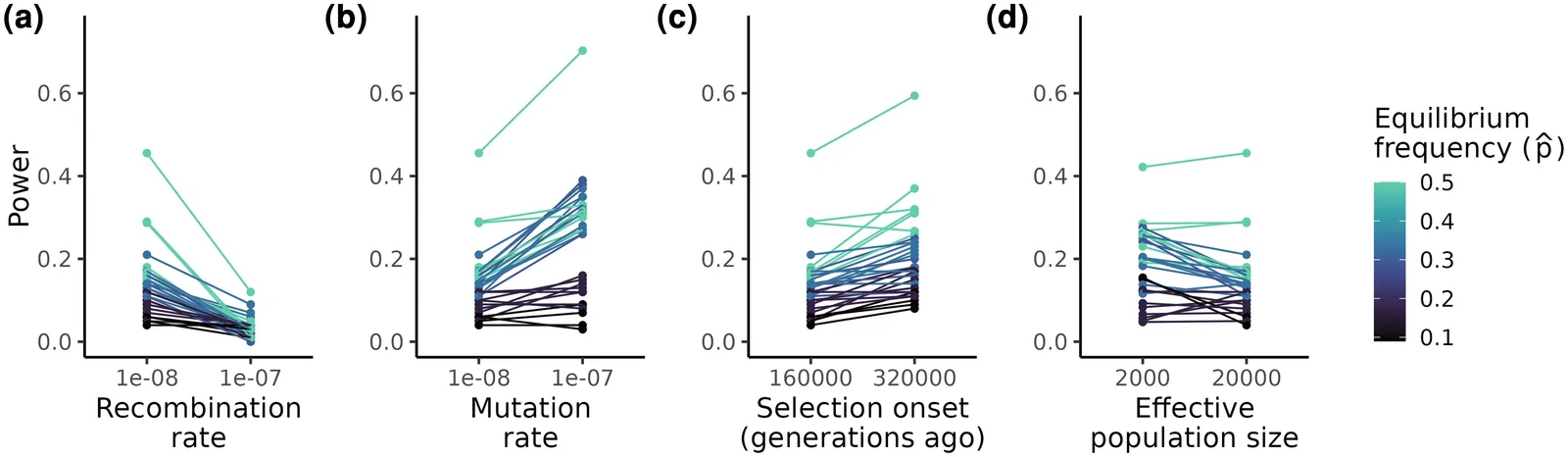
Power to detect balancing selection in our reference set of simulations (points on the left of panels a–c and on the right of panel d) compared to simulations with increased recombination rate, mutation rate, age of the selected allele, and reduced effective population size. Balancing selection was simulated under overdominance, and parameters in the reference set of simulations are *N_e_* = 20,000, recombination and mutation rates of 10^−8^ per site per generation, and selection onset time 160,000 generations ago.

**Table 3:** Summary of statistical results for the successful detection of balanced loci as a function of simulated parameters.

Predictors included in the model	Predictors discussed in main text	Deviance	% deviance explained	Significance (*P*-value)	Slope (*b* value)
Equilibrium frequency ($\hat{\;p}$), selection strength (*α*), log_10_ of **recombination rate (*r*)**	*r*	215.80	33.84%	<0.001	−1.168
Equilibrium frequency ($\hat{\;p}$), selection strength (*α*), log_10_ of **mutation rate (*µ*)**	*µ*	22.81	2.31%	<0.001	0.016
Equilibrium frequency ($\hat{\;p}$), selection strength (*α*), **allelic age (*T*)** in *N_e_* generations	*T*	508.89	29.34%	<0.001	0.0815
Equilibrium frequency ($\hat{\;p}$), Selection strength (*α*), **Effective population size (*N_e_*)** in thousands	*N_e_*	15.16	2.05%	<0.001	0.153
	*N_e_* * $\hat{\;p}$	35.80	4.85%	<0.001	−0.321

Only terms including the variable of interest (marked in bold on the first column) and with *P*-value <0.05 and percentage of deviance explained > 1% are shown. Full results are provided in the Supplementary Results.

The intensity of the signal of balancing selection also depends on the timespan over which a balanced polymorphism has been maintained, with the age of the balanced polymorphism in our simulation data having a positive effect on our power to detect it (Fig. 5c; Tables 3, S5, S11, S17, S23). For every additional *N_e_* generation of the age of the balanced polymorphism, the odds of successfully detecting balancing selection increase by 8.5%.

Finally, increasing the effective population size generally increases the detectability of balanced polymorphisms, though this effect is restricted to polymorphisms with lower equilibrium frequency (Fig. 5d). The binomial GLM reveals a significant positive effect of *N_e_* on power (Table 3), with a significant negative interaction with the equilibrium frequency (Table 3). Thus, the model predicts that among loci with a minor allele equilibrium frequency of $\hat{\;p}=0.3$, a 10-fold increase in *N_e_* increases the odds of successfully detecting balancing selection by over 70%. In contrast, for loci with an intermediate equilibrium frequency of $\hat{\;p}=0.5$, the same increase in *N_e_* would reduce the odds of detection by 7%. We note, however, that these effects of *N_e_* on detection power are weak and not consistent across detection methods (Supplementary Results).

## Discussion

We assessed the propensity of balancing selection to maintain long-term stable polymorphism and the power of current inference methods to successfully detect such loci. The main message from our analyses is that detecting balanced sites in population genomic data is difficult, at least in the populations of constant size that we simulate here. Even where balancing selection is strong relative to drift and has maintained allelic variation at frequencies closely clustered around an equilibrium frequency of 0.5 for time spans that vastly exceed neutral coalescent times (such as 16*N_e_* generations), state-of-the-art methods of inference still only rarely exceed a power of 50% (Figure S13). For example, an OD polymorphism with relatively strong and symmetrical selection (*s*_1_ = *s*_2_ = 0.1, resulting in $\hat{\;p}=0.5$) that is 8*N_e_* generations old is detected as a balanced locus with a probability of only 29% in our reference simulations. These selection coefficients are of the same order of magnitude as the ones estimated for the remarkable example of net overdominance maintaining horn size polymorphism in Soay sheep (Johnston et al. (2013), see their Table S3). While this locus was identified with the help of phenotypic data, our results thus show that similar loci would be missed in the majority of cases when analyzing genomic data alone. This finding contrasts with the notion that inference power with current approaches is good (Bitarello et al. 2023), a perception that is probably due to the empirical focus on human populations with their particular demographic histories (see below).

While power in our simulations is moderate in the best of cases, it further decreases under less ideal and perhaps more realistic conditions. The selection parameters have particularly pronounced effects on the long-term maintenance and eventual detectability of a balanced locus. Selection and dominance coefficients determine the equilibrium frequencies of balanced polymorphisms (and hence whether polymorphisms under AP or SA will be subject to balancing selection at all) and the efficacy with which selection maintains the alleles near equilibrium. Each of these factors influences the long-term stability of a balanced polymorphism. But even when a polymorphism is long-term stable, balancing selection resulting in allele frequency equilibria close to zero or one are unlikely to be identified, because polymorphisms produce weak signals of balancing selection.

### Model of selection mainly affects maintenance

The models of selection differ strongly in the strength of selection they generate and hence their propensity to maintain polymorphism in the first place. With OD, the net strength of selection is a simple linear function of its selection coefficients ($\alpha =s_{1}+s_{2}$), leading to effectively strong net selection in cases where population-scaled selection parameters are large (ie $N_{e}s_{1}\gg 1$ and $N_{e}s_{2}\gg 1$). In contrast, for scenarios of AP and SA without dominance reversals, the net strength of balancing selection (when it occurs) depends on the product of the selection coefficients (ie $\alpha =\frac{1}{2}s_{1}s_{2}$ when *h* = 0.5), so that the same values of $s_{1}$ and $s_{2}$ lead to effectively weaker balancing selection under AP and SA than under OD (see Appendix S2 for derivations of equations for *α*). Assuming the distribution of fitness effects is similar under the 3 mechanisms, then loci subject to antagonistic selection should be less likely to remain polymorphic over extended periods of time. While dominance reversals dampen the differences between antagonistic selection scenarios and OD, they only vanish when dominance reversals are complete.

While the specific model of balancing selection has a strong effect on the propensity to maintain balanced polymorphisms, it did not have an overall effect on detection power with NCD. However, our analyses revealed more subtle interactions between the model of selection, the net strength of selection (*α*), and the equilibrium frequency ($\hat{\;p}$). Specifically, while power generally increases with the strength of selection, selection strength has little or no effect on power under OD and AP when equilibrium frequencies are low (eg 0.1 and 0.25 in Fig. 4). These effects are surprising, given that *α* is intended to capture the strength of selection in a way that is independent of the mechanism generating it. We note, however, that the expressions for *α* are approximations based on the assumption of effectively weak selection (see Appendix S1) and interaction effects might be due to higher-order terms that were omitted. Detection power with BalLeRMix differed significantly between selection models in both run modes (equilibrium frequency optimized or fixed to true value), but the direction of the effect was not consistent between run modes (Supplementary Results), making it difficult to draw any general conclusion about the effects of selection model on BalLeRMix power.

### Selection onset and effective population size

Our simulations also demonstrate that the probability of inferring balancing selection at a locus increases with the age of the polymorphism, as signatures of linked variation build up over time. Importantly, the timescale of this process is linked to the effective size of the population. For the signature of selection in linked variation to be discernible, the coalescent time at the balanced site needs to substantially exceed the average neutral coalescent time of 4*N_e_* generations. This means that when *N_e_* is larger, balanced polymorphisms need to be older, in generations, to be detectable. Likewise, for a given age in generations, polymorphisms are harder to detect in species with high *N_e_* than in those with low *N_e_*.

The timescales over which signatures of selection accumulate also matter for the presence of balancing selection more generally, and how it differs between species. The time required for signatures to build up can be considerable in geological time. In humans, for example, an age of 8*N_e_* generations corresponds to roughly 4My (based on a historical *N_e_* of $2\times 10^{4}$ and a 25-year generation time), or roughly 2 thirds of the time since the human–chimpanzee split. The requirement for selection to remain more or less constant over these extended periods of time could substantially limit the prospects for the presence of long-term balancing selection not only in humans but even more so in species with large *N_e_*. The effect is somewhat attenuated by the fact that generation times tend to correlate negatively with *N_e_*. An analysis of the relationship between *N_e_* and generation time (*g*) (Chao and Carr 1993) suggests that, very roughly, $g\propto N_{e}^{-0.35}$, in which case the geological timescale (in years) that is required to generate a genomic signal of long-term balancing selection is on the order of $N_{e}g\propto N_{e}^{0.65}$. Thus, despite the inverse relationship between $N_{e}$ and *g*, the timescale for generating a signal of balancing selection should generally be higher in lineages with larger *N_e_* and shorter *g* (such as insects). As a consequence, for a given geological age of a polymorphism, the signals of balancing selection are less likely to be visible in large-*N_e_* lineages because the criteria for “long-term balancing selection” (as defined in Bitarello et al. 2023) are more restrictive.

Interestingly, *Drosophila melanogaster* may be an exception to this broader pattern, in that it has a short generation time (∼15 generations/year or 0.07 years/generation; Turelli and Hoffmann 1991) for its effective population size (an historical *N_e_* of roughly 10^6^; Arguello et al. 2019). Based on these figures, 8*N_e_* would correspond to 0.56My, much shorter than the equivalent time in humans, which makes it more likely that broader environmental conditions remain constant enough to allow for the maintenance of balanced polymorphisms in this species. In addition, extremely short generation times such as those in *Drosophila* provide further scope for balancing selection due to seasonal fluctuations in the environment (Wittmann et al. 2023), which is an unlikely avenue for balancing selection in species with longer generation times.

The effective population size also has other direct and indirect effects on the presence and detectability of balanced polymorphisms. Large *N_e_* leads to reduced drift and hence more effective selection to maintain balanced polymorphisms; all else being equal, it should thus increase the proportion of balanced loci that remain long-term polymorphic. Yet, the effects of *N_e_* on power to detect balanced polymorphisms are weak and inconsistent across methods (Supplementary Results). We believe this is due to the opposing effects that *N_e_* could have on power. On the one hand, higher *N_e_* may favor detection by reducing genetic drift, thus maintaining polymorphisms closer to their equilibrium frequencies. This may particularly facilitate detection of balanced loci whose equilibria are further from the target frequency of 0.5 used for NCD calculations. On the other hand, *N_e_* also has adverse effects on the signatures at linked neutral polymorphisms. Species with large *N_e_* tend to have larger effective recombination rates (larger $N_{e}r$), owing trivially to the effect of larger *N_e_* but also due to the fact that large-*N_e_* species often have relatively streamlined genomes and accordingly higher per-nucleotide recombination rates (*r*) (Lynch 2006). Elevated recombination will erode the balancing selection signal of intermediate frequencies at neutral sites that are closely linked to selected variants and thereby make the signature sparser and inference harder. This effect can be compensated by a higher mutation rate (*µ*), which favors the generation of linked polymorphism around the balanced site. Thus, for a given *N_e_*, the power to detect balancing selection is determined by the ratio of mutation to recombination rate, *µ*/*r*.

### Demographic history and comparison to previous power estimates

In relation to the discussion of *N_e_* above, it is important to note that we have carried out simulations in populations of constant size. Changes in the effective population size over time, such as sudden contractions (bottlenecks) and subsequent expansions, can improve detection. Thus, while increased drift during a recent population bottleneck may lead to random loss of some balanced polymorphisms, those that survive stand out more clearly from a neutral background with a high rate of recent coalescence (a more recent average TMRCA) and hence a more leftward-shifted SFS. It is likely that this phenomenon has facilitated the identification of balanced polymorphic loci in humans, where migration out of Africa has resulted in a series of bottlenecks and founder events that have systematically shifted the neutral SFS.

More complex scenarios of past changes in *N_e_*, including oscillations and the combined effect of changes in population structure and migration, could have varied effects on the maintenance and detectability of balanced polymorphisms that will depend on the specific scenario. For example, in *Drosophila melanogaster*, dramatic seasonal fluctuations in census size, and hence *N_e_*, have been reported (Nunez et al. 2024). The effect of these regular demographic changes on balanced polymorphisms and background neutral coalescence will depend on the amplitude of these fluctuations, which determines the proportion of lineages that coalesce during the phases of low *N_e_*. Given the frequency and regularity of seasonal fluctuations relative to the timespans that balanced polymorphisms need to be maintained to be detectable, their effect on balanced polymorphisms and their genomic signatures will be well captured by the long-term *N_e_* of the population. But this might be different for other complex demographies, which could affect both the maintenance of balanced polymorphisms and their detectability in different ways.

The small *N_e_* and specific demographic history of humans likely explain why previous analyses of the power of current inference methods have provided higher estimates of power (reaching over 80% under some conditions) than the ones we report here for our human-like “reference” parameters. While methods like NCD or BalLeRMix are general in scope, they have typically been benchmarked using simulated data that follow a demographic history and a mutation-to-recombination rate ratio that is characteristic of human populations (eg 2.5 in Bitarello et al. (2018) and sampled between 2.5 and 0.83 in Cheng and DeGiorgio (2020)), and relatively old polymorphisms (eg up to 5 million years in Bitarello et al. (2018) and 650,000 generations with *N_e_* = 10^4^ in Cheng and DeGiorgio (2020)—16.25 million years with Bitarello et al.’s (2018) generation time of 25 years). Given the specific assumptions made in the original method publications and the substantial variation in power as a function of the parameters used, we reiterate that it is imperative to combine any application of existing methods with a careful power analysis, parameterized as much as possible to fit the study organism and population (Bitarello et al. 2018, 2023).

### Methodological notes on NCD

We have deliberately focused our main analyses on a single approach, NCD, to evaluate how different population genetic parameters determine the efficacy of balancing selection and the signals it generates. Other methods to detect balancing selection, including BALLET (DeGiorgio et al. 2014), BetaScan (Siewert and Voight 2017), BalLeRMix (Cheng and DeGiorgio 2020) or even the HKA test (Hudson et al. 1987), essentially exploit the same or similar signals in the data (ie an excess of polymorphic sites, often with alleles segregating at intermediate frequencies; see Bitarello et al. (2023) for a review, and hence will be affected similarly by the parameters we explore (see Supplementary Results for additional analyses with BalLeRMix). It is not our aim to compare different methods, but we will briefly discuss a few specificities of our application of NCD that are expected to have small effects on the absolute power we estimate.

First, in our main analyses, we use 0.5 as the NCD “target frequency” (ie the frequency value from which NCD measures the mean squared distance of allele frequencies in a window). This represents typical usage in applications to empirical data, where the target frequency is not known, and is therefore set to an arbitrary intermediate value. Previous analyses (Bitarello et al. 2018), however, have shown that power is minimally impacted by the exact target frequency, and we repeated our analyses setting the target frequency to the true equilibrium frequency (calculated from simulated selection coefficients), and found that power is similarly affected by the same predictors (Figure S14 and Supplementary Results).

Second, we use NCD with a fixed window size of 3kb, independently of the recombination rate. In cases where the recombination rate is high and regions of elevated polymorphism are narrow, calculating the SFS over a smaller interval can improve power, although this is offset by the fact that the small numbers of SNPs in a narrow focal window generate noise that negatively impacts the inference (Figure S15). Again, real-world applications should be preceded by power analyses that explore these effects and can help to optimize the analysis.

Finally, we use a variant of NCD here, NCD2, that also incorporates fixed differences to an outgroup into the SFS (as does BalLeRMix's B2). This can improve power by effectively shifting the SFS at neutral regions further leftwards, thanks to mutations that fix over the timespan where neutral loci have already coalesced, but balanced loci remain polymorphic (Figure S16, shaded area). In empirical applications, these informative mutations are included at the price of added noise that arises from the variable numbers of fixed differences that arose at older times or on the branch toward the outgroup (Figure S16, red dashed branches) and that contribute equally to the SFS of neutral and balanced loci. We note that our simulation approach does not include an outgroup, and it has a recapitation stage where fixed differences do not arise (see Methods). It therefore does not incorporate the latter, uninformative type of fixed differences; at the same time, it includes most but not all of the former, informative ones (it includes ∼68% of these for *T* = 8*N_e_* and ∼87% for *T* = 16*N_e_*). Accordingly, in our simulations, the benefit of including fixed differences is not fully maximized —though the maximum benefit of fixed differences is rarely realized in empirical applications, where divergence to the outgroup cannot be precisely chosen to minimize the inclusion of uninformative fixed differences.

### General considerations on differences between species

Our analyses make it clear that the prospects of identifying targets of balancing selection are expected to vary significantly and systematically among species. Thus, species will harbor variable numbers of long-term balanced loci, depending on the effective sizes of their populations. Furthermore, the effective size, combined with mutation and recombination rates, will determine how difficult these sites are to identify as targets of balancing selection. In humans, eg, the small effective population size means that only a limited number of balanced polymorphisms are expected to have remained polymorphic over sufficiently long periods of time. On the other hand, the same low effective population size, together with the high ratio of mutation to recombination rate ($\frac{\mu }{r}=\frac{1.2\times 10^{-8}}{1.3\times 10^{-8}}\approx 1$ (Pritchard 2023 Chap. 1.1)), favor detection of such loci. In contrast, *D. melanogaster*, and possibly other insects, presents a much harder case for identification, as the recombination rate is high relative to the mutation rate ($\frac{\mu }{r}=\frac{3\times 10^{-9}}{3\times 10^{-8}}=0.1$ (Wang et al. 2023)), which our analyses suggest should reduce power by roughly 5-fold compared to a species with human-like mutation and recombination parameters (mean power across simulated parameters in Fig. 5a, 0.11 vs. 0.02).

Of course, as discussed above, there are other important factors at work that affect the detection of balanced polymorphic loci in the 2 species, such as the time required for long-term balancing selection, the scope for seasonal selection, effects of *N_e_* on the maintenance of polymorphism on the one hand and its detectability on the other, and species-specific demographic histories. Overall, there is little doubt that the detection of balancing selection in *Drosophila* is challenging, which suggests that there could be many undetected targets of balancing selection in this and similar species. Indeed, a genome-wide screen for balancing selection in fruit flies combining Theta and Tajima's *D* identified roughly 100 candidate balanced loci (Croze et al. 2017), compared to over 1,000 candidates in humans (Bitarello et al. 2018). The notion that large numbers of undetectable balanced polymorphisms might exist is in line with the high levels of standing genetic variation for major fitness components in this species, which exceeds the variation explainable under mutation-selection-drift balance (Charlesworth 2015; Sharp and Agrawal 2018). While it seems plausible that at least some of these balanced polymorphisms would be detected if detection methods were more powerful, others could remain invisible if they are relatively young (relative to 4*N_e_*). It is difficult to assess these alternatives in the absence of more powerful approaches, and similar conclusions may apply to many other species with equally unfavorable population parameters.

## Materials and methods

### Population genetic simulations of balancing selection

We used simulations to assess which regions of the parameter space generate genomic signatures of balancing selection that can be detected using current approaches. Simulations were run in SLiM (version 4.0.1, (Haller et al. 2019)), a forward simulation framework for population genetics. In line with the analytical derivations above, we implemented overdominance (OD), antagonistic pleiotropy (AP), and sexually antagonistic selection (SA), in addition to simulations under neutrality. In the simulations with selection, we simulated a single focal site, embedded centrally in a chromosome of 10,000 bp, large enough for neutral sites in the periphery to be unaffected by selection at the central site. To implement the 3 selection mechanisms, we used custom functions (see SLiM scripts available at https://github.com/reuterlab/bls_sim/) to run a non-Wright-Fisher model for populations of constant size with nonoverlapping generations. In each generation, the code generated offspring by randomly drawing parents based on their fitness values, calculated according to their genotype at the locus under selection and the selection mechanism and parameters (selection coefficients, dominance, cf. Table 1) being simulated. We used the same code for reproduction in the neutral simulations, but set the fitness of all individuals to 1. In all simulations, the default sex ratio of 1:1 was used.

At the start of each simulation, a mutation at the focal site was introduced on a single chromosome, randomly chosen within the population. The mutation would be of type A_1_ or A_2_ (see Table 1, eg either male- or female-beneficial for SA) with equal probability. To reduce the number of simulation runs where the polymorphism was quickly lost due to drift when rare, we initially subjected the mutant allele to directional positive selection with *s* = 0.5 and *h* = 1/2 until the mutant allele frequency reached 5% (or the mutant allele was lost). This selective boost facilitates the establishment of the polymorphism, but due to its old age and short duration should have negligible influence on the patterns of linked variation. For polymorphisms that reached a frequency of 5%, simulations then switched from directional to balancing selection with a specified mechanism and parameter combination. We recorded the frequency of the mutant (derived) allele and the genealogy of all chromosomes in the population at 16,000, 32,000, 160,000 and 320,000 generations.

To optimize the runtime of simulations, we did not include neutral mutations in any of the SLiM simulations. Instead, we recorded the full chromosome-wide genealogies from each simulation (using “tree-sequence recording”, (Haller et al. 2019)). We then used “recapitation” in pyslim (version 1.0.4, (Ralph et al. 2023)) to generate the preceding genealogical relationships of the starting chromosomes up to their most recent common ancestor. Finally, we added neutral mutations to the complete recapitated genealogies with msprime (version 1.2.0, (Baumdicker et al. 2022)).

The parameter values used in our simulations are summarized in Table 4. We explored selection and dominance coefficients to match the analytical results. For the species-specific parameters of effective population size ($N_{e}$), recombination rate (*r*), and mutation rate (*µ*) were chosen to cover a broad range of relevant scenarios. Thus, we simulated population sizes of *N* = $N_{e}$ = 2,000 and *N* = $N_{e}$ = 20,000 and neutral mutation at rates of *µ* = 10^−7^ and *µ* = 10^−8^ per base per generation, such that the expected genetic diversity ($\pi =4N_{e}\mu$) ranges from 8 × 10^−5^ to 8 × 10^−3^. The highest expected genetic diversity (for $N_{e}$ = 20,000 and µ = 10^−7^) approaches that of *Drosophila melanogaster* (*π* = 10^−2^ (Leffler et al. 2012)), while the combination of $N_{e}$ = 20,000 and *µ* = 10^−8^ generates diversity similar to that estimated in humans (*π* = 8 × 10^−4^ (Pritchard 2023 Chap.1.1)). We simulated recombination rates of *r* = 10^−7^ and *r* = 10^−8^ per base per generation to include combinations of *µ* = *r* = 10^−8^, which are similar to the values estimated in humans (Pritchard 2023 Chap. 1.1), as well as a ratio of mutation to recombination rate similar to that estimated in *Drosophila melanogaster* (*µ*/*r* = 10^−8^/10^−7^ = 0.1, (Wang et al. 2023)) and a scenario where mutation rate exceeds recombination rate (*µ*/*r* = 10^−7^/10^−8^ = 10), which should facilitate the detection of signatures of selection. Finally, we used 2 values for the onset of selection (or age of the polymorphism), *T* = 8*N_e_* and *T* = 16*N_e_* (16,000 and 32,000 generations for *N_e_* = 2,000 and 160,000 and 320,000 generations for *N_e_* = 20,000). These values roughly correspond to the definitions of long-term and ultra-long-term balancing selection (Bitarello et al. 2023). Both significantly exceed the expected coalescence time (time to the most recent common ancestor, TMRCA) under neutrality$4N_{e}$. The probability of observing a TMRCA larger than 8*N_e_* generations under neutrality is approximately 0.054, while that of a TMRCA larger than 16*N_e_* generations is approximately 0.001 (Wakeley 2009, p. 77, Eq. 3.27).

**Table 4:** Parameter values used in the simulations.

Selection model	Overdominance (OD) Antagonistic pleiotropy (AP) Sexual antagonism (SA)
Selection coefficients (*s*)	*s_weak_* = {0.01, 0.02,0.05,0.1} *s_strong_* = {0.1,0.2,0.5,1}
Dominance coefficients (*h*, only defined for AP and SA)	0.25 0.5
Time of onset of selection (*T*, in generations)	**8*N_e_*** 16*N_e_*
(Effective) population size (*N*=$N_{e}$)	2,000 **20,000**
Recombination rate (*r*)	10^−7^ **10^−8^**
Mutation rate (*µ*)	10^−7^ **10^−8^**

We highlight in bold the values used in our “reference” set of simulations, which approximate the parameter values estimated in the literature for humans.

To explore the effect of the strength of selection, equilibrium frequency and model of selection on the maintenance of the selected polymorphism (Figures S1–S6) and the inference of balancing selection (Fig. 3), we ran simulations for a reference set with human-like parameters (bold values in Table 2, *N_e_* = 20,000, *µ* = *r* = 10^−8^, *T* = 8*N_e_* =160,000 generations) under OD, AP, and SA across combinations of selection coefficients *s_i_* and *s_j_* drawn from two 4-by-4 grids with weaker selection {0.01,0.02,0.05,0.1} and stronger selection {0.1,0.2,0.5,1.0}. To explore the effects of mutation rate, recombination rate, selection onset time and *N_e_*, (Fig. 5), we ran similar sets of simulations for OD only, but changing the values of each of these parameters relative to the reference set of simulations (ie *µ* = 10^−7^, *r* = 10^−7^, *T* = 16*N_e_* = 320,000 generations, *N_e_* = 2,000).

In simulations to assess the maintenance of polymorphisms, we ran 100 replicate simulations in which the mutation successfully established (*ie* reached 5%) for each parameter combination. To assess the power to detect signatures of balancing selection, we complemented these with additional simulations to obtain sets of 100 replicate simulations for each parameter combination in which the polymorphism was maintained.

### Power of inference of balancing selection

We used the NCD (noncentral deviation) method (Bitarello et al. 2018) to infer balancing selection on the simulated data (but for complementary analyses using BalLeRMix (Cheng and DeGiorgio 2020), see Methods and Supplementary Results). Like most other current inference methods, NCD detects regions under balancing selection due to their signal of excess intermediate-frequency polymorphism. NCD for a genomic window with *n* informative sites is defined as:


$$NCD(tf)=\sqrt{\frac{\sum_{i=1}^{n}\;(\;p_{i}-tf{)}^{2}}{n}}$$

where $p_{i}$ is the allele frequency at the *i*th informative site, and *tf* is the target frequency (set as an intermediate value eg 0.5). The presence of a balanced site in the focal region with allele frequencies close to the target frequency then results in a low value of the NCD statistic, compared to a region under neutrality or purifying selection.

In our application of this approach, we used a refined version of the statistic, NCD2. This statistic gains power by also considering fixed differences (relative to an outgroup) in the site frequency spectrum (Bitarello et al. 2018), thus exploiting the signal of increased polymorphism and lower rate of substitution in regions under balancing selection. The mutations that contribute to power here are those that fix over the timespan where neutral loci have already coalesced, but balanced loci remain polymorphic (Figure S16, shaded area), while those that are older or occur on the outgroup branch are not affected by balancing selection and contribute mostly noise (Figure S16, dashed red branch). We note that our simulation approach without an outgroup exclusively captures the most informative fixed differences (Figure S16, solid light red branch); it does so through mutations that become fixed in forward neutral simulations that fully coalesce (95.4% of simulations of 8*N_e_* generations, 99.7% for 16*N_e_*, average coalescence time ∼4*N_e_* generations). However, we miss a proportion of mutations that would have occurred under neutrality in the time taken for simulations with selection to coalesce during the recapitation stage (on average 1.8*N_e_* generations, Figure S16 shaded gray area).

To assess the statistical significance of the NCD2 values, we generated a null distribution by running 1,000 neutral simulations for each combination of relevant parameters (*N_e_*, *µ* and *r*) and calculating NCD2 for each. We then calculated a *P*-value for an NCD2 value from a simulation with selection by determining the proportion of NCD2 values from the null distribution (with corresponding *N_e_*, *µ*, and *r*) that was lower than the NCD2 value with selection. Power was then calculated as the proportion of the 100 simulations generated under a particular set of parameters for which *P* ≤ 0.01.

### Statistical analysis of power estimates

We fitted binomial Generalized Linear Models (GLMs) to our power data to directly model the probability of detecting or failing to detect balancing selection as a function of the parameters that were varied in our simulations. The models used a logit link function and were fitted to the number of successful and unsuccessful instances of detection as the response variable. The significance of model terms was determined with an analysis of deviance, a series of sequential likelihood ratio tests based on the Chi^2^ distribution. This analysis further makes it possible to assess the importance of each model term through its deviance (the amount of variability in the response explained by a model term) relative to the null deviance, the total amount of variability in the response values analyzed. We note that all models showed a degree of overdispersion, as indicated by the residual deviance exceeding the number of residual degrees of freedom several-fold. However, running alternative models that accommodate overdispersion (as a “quasibinomial” model, combined with F-tests) produced qualitatively identical results.

These GLMs fit a linear (additive) model of detection probability (π) as a function of the simulation parameters. The model is fitted on the log-odds scale (where odds are π/(1−π)) that accommodates the fact that probabilities are bounded by 0 and 1. The estimated model parameters, back-transformed to the odds-scale, provide information on the proportional change in the detection probability that is independent of the value of π (while the change in the actual probability due to a change in a predictor variable changes over the range of π).

## Supplementary Material

msag228_Supplementary_Data

## Data Availability

All simulation and analysis code and data for the statistical analysis of power are available on Github at https://github.com/reuterlab/bls_sim/. Simulated genetic data (VCF files) are available on Zenodo at DOI 10.5281/zenodo.21512158.
