# Supplementary material for: Characterizing the detectable and invisible fractions of genomic loci under balancing selection
Source: Mol Biol Evol. 2026 Oct 5;43(10):msag228. doi: 10.1093/molbev/msag228 (PMC13635458; doi:10.1093/molbev/msag228)
Supplement: Supplementary file 1 [file msag228_supplementary_data.zip › BrandtEtAL_Appendices.pdf]

Appendices — Characterising the detectable and invisible fractions of genomic loci under balancing selection

Débora Y. C. Brandt, Aida M. Andrés, Tim Connallon and Max Reuter

Appendix 1 – Conditions for internal frequency equilibria

We model balancing selection making three simplifying assumptions that should apply broadly to many (perhaps most) cases where this type of selection is present. First, we focus on populations of diploid, randomly mating (outbred) individuals (see Glémin (2021) for a comprehensive treatment of balancing selection in self-fertilizing species). Second, we consider loci with two major allele types, which we label A_1 (at a population frequency of p) and A_2 (frequency $1 - p$). Third, in scenarios involving antagonistic selection (i.e., antagonistic pleiotropy and sexual antagonism), we only consider scenarios of dominance that are amenable to balanced polymorphism, i.e. cases where each fitness component or sex either lacks dominance (fitness effects are additive in each context of selection) or there is a beneficial reversal of dominance, in which the costs of expressing the "wrong" allele for a given context of selection are partially or completely recessive (reviewed in Connallon and Chenoweth 2019; Grieshop et al. 2024). Our exposition draws upon previous studies of balancing selection theory (*e.g.*, Robertson 1962; Kidwell et al. 1977; Curtsinger et al. 1994; Connallon and Clark 2013), with Table 1 providing an outline of the fitness values associated with each genotype and for each model of selection.

Parameters for the three models fall within the range $1 \geq s_1, s_2 > 0$. Dominance coefficients are within the range $0.5 \geq h \geq 0$, which includes additive effects (h = 0.5) and beneficial reversals of dominance ($0.5 > h$). Note that many of our analyses, we focus on scenarios where fitness effects are small, *i.e.*.: $1 \gg s_1, s_2 > 0$.

Overdominant selection (OD)

With OD, the heterozygotes for the locus (A_1A_2) have the highest fitness and A_1A_1 and A_2A_2 homozygotes suffer fitness reductions of s_1 and s_2, respectively (Tab. 1). Evolutionary dynamics are captured by the following difference equation describing allele frequency change across a single generation:

$$\Delta p = \frac{(s_1 + s_2)p(1-p)(\hat{p} - p)}{\bar{w}} \approx (s_1 + s_2)p(1-p)(\hat{p} - p) \quad \text{(A1)}$$

where $\bar{w} = p^2(1 - s_1) + 2p(1-p) + (1-p)^2(1 - s_2)$ denotes mean fitness and:

$$\hat{p} = \frac{s_2}{s_1 + s_2} \quad \text{(A2)}$$

is the equilibrium frequency for the A_1 allele. The final approximation in eq. (A1) applies under weak selection ($0 < s_1$, $s_2 << 1$). Note that the entire range of parameter values in this model generates balancing selection (*i.e.*, $0 < \hat{p} < 1$ for the plausible parameter range, $0 < s_1, s_2 < 1$).

Antagonistic pleiotropy (AP)

Following previous models (e.g. Curtsinger et al., 1994; Rose, 1982), we consider a locus with antagonistic pleiotropic effects on two major fitness components. Selection is directional within each component (Tab. 1) and fitness components combine multiplicatively to determine overall fitness. The evolutionary dynamics are described by:

$$\Delta p = \frac{\left((s_1 + s_2)(1 - 2h) + 2s_1 s_2 h^2\right)p(1-p)(\hat{p} - p)}{\bar{w}}$$

$$\approx \left((s_1 + s_2)(1 - 2h) + 2s_1 s_2 h^2\right)p(1-p)(\hat{p} - p) \quad \text{(A3)}$$

where $\bar{w} = p^2(1 - s_1) + 2p(1-p)(1 - s_1 h)(1 - s_2 h) + (1-p)^2(1 - s_2)$ and the polymorphic equilibrium (whenever it is valid) is:

$$\hat{p} = \frac{s_2(1-h) - s_1 h + s_1 s_2 h^2}{(s_1 + s_2)(1 - 2h) + 2s_1 s_2 h^2} \quad \text{(A4)}$$

The equilibrium is valid under the following condition:

$$\frac{s_1 h}{1 - h + s_1 h^2} < s_2 < \frac{s_1(1-h)}{h(1 - s_1 h)} \quad \text{(A5)}$$

and otherwise, selection on total fitness is directional and favours fixation of either A_1 or A_2.

Sexually antagonistic selection (SA)

Following Kidwell et al. (1977), sexually antagonistic alleles are under opposing directional selection in males and females, characterised by a selection and dominance coefficient for each sex (Tab. 1). Letting $p = (p_f + p_m)/2$ represent the overall frequency of A_1 in the population, where p_f and p_m represent the respective frequencies in female and male gametes contributing to fertilization, s_1 and s_2 represent the selection coefficients against alleles A_1 and A_2 (respectively), and for the case where allele A_2 is selected against in females, the exact allele frequency dynamics are given by $\Delta p = \frac{1}{2}\left(\Delta p_f + \Delta p_m\right)$, where:

$$\Delta p_f = \frac{p_m p_f + (p - p_m p_f)(1 - s_2 h)}{p_m p_f + 2(p - p_m p_f)(1 - s_2 h) + (1 - p_f)(1 - p_m)(1 - s_2)} - p_f \quad (A6a)$$

and

$$\Delta p_m = \frac{p_m p_f (1 - s_1) + (p - p_m p_f)(1 - s_1 h)}{p_m p_f (1 - s_1) + 2(p - p_m p_f)(1 - s_1 h) + (1 - p_f)(1 - p_m)} - p_m \quad (A6b)$$

Within the range $0 \leq h \leq 0.5$ which we consider here, and assuming modest to weak selection ($0 < s_1, s_2 << 1$), the allele frequency dynamics are closely approximated by:

$$\Delta p \approx (s(1 - 2h) + 2h^2 s_1 s_2) p(1 - p)(\hat{p} - p) \quad (A7)$$

(Connallon and Clark 2013), where $s = \frac{1}{2}(s_1 + s_2)$, and $\hat{p}$ represents the polymorphic equilibrium when valid. With no dominance in either sex (h = 0.5), the polymorphic equilibrium frequency (when it exists) is $\hat{p} = \frac{(s_2 - s_1 + s_1 s_2)}{2 s_1 s_2}$, and for cases involving dominance reversals (h < 0.5), the polymorphic equilibrium is $\hat{p} \approx \frac{(s_2(1-h) - s_1 h)}{(s_1 + s_2)(1 - 2h)}$. The condition favouring a polymorphic equilibrium (based on the exact eqs. (6a-6b)) is:

$$\frac{s_1 h}{1 - h + s_1 h} < s_2 < \frac{s_1 (1 - h)}{h(1 - s_1)} \quad (A8)$$

(Kidwell et al. 1977; Connallon and Chenoweth 2009).

If we assume that each selection coefficient is uniformly distributed with a mean of $\bar{s} = \frac{1}{2} s_{\max}$ (where $s_{\max}$ represents the maximum selection coefficient, $0 < s_{\max} \leq 1$), then the proportion of parameter space that results in balancing selection under AP will be:

$$\begin{aligned} f_{bal} &= 1 - \frac{2h^2 s_{\max} + 2(1 - h) \log\left(\frac{1 - h}{1 - h + h^2 s_{\max}}\right)}{s_{\max}^2 h^3} \\ &= \frac{1 - 2h}{1 - h} + \frac{4h^3 \bar{s}}{3(1 - h)^2} + O(s_{\max}^2) \end{aligned} \quad (A9)$$

while that that results in balancing selection under SA is:

$$\begin{aligned} f_{bal} &= 1 - \frac{2h s_{\max} + 2(1 - h) \log\left(\frac{1 - h}{1 - h + h s_{\max}}\right)}{s_{\max}^2 h} \\ &= \frac{1 - 2h}{1 - h} + \frac{4h^2 \bar{s}}{3(1 - h)^2} + O(s_{\max}^2) \end{aligned} \quad (A10)$$

Appendix 2 - Criteria for maintaining a long-term balanced polymorphism

A long-term polymorphism requires balancing selection to be both consistent over time and strong enough to offset the tendency of genetic drift to eliminate the polymorphism. Provided

balancing selection is temporally consistent, then its capacity to maintain polymorphism can be quantified using standard diffusion theory (Rice, 2004; Wright, 1945). In the models presented above, the expected change in allele frequencies at a single locus under balancing selection (with change measured across a single generation) is:

$$M = \alpha p(1-p)(\hat{p}-p) \qquad \text{(A11)}$$

where p again refers to the frequency of A_1, $\hat{p}$ is the equilibrium under balancing selection, and α represents a model-specific constant that reflects the strength of balancing selection (these can be extracted from eqs. (1), (3) and (7), which yield: $\alpha = s_1 + s_2$ for the overdominance model, $\alpha = (s_1+s_2)(1-2h) + 2s_1s_2h^2$ under antagonistic pleiotropy, and $\alpha = s(1-2h) + 2s_1s_2h^2$ under sexual antagonism, where $s = \frac{1}{2}(s_1+s_2)$). The variance in allele frequency change owing to drift is $V = \frac{p(1-p)}{2N_e}$, where N_e is the effective size of the population. Ignoring effects of recurrent mutation, the stationary distribution loci subject to balancing selection (*i.e.*, the long-term probability density function for allele frequency p) is:

$$f(p) = \frac{C}{p(1-p)}\exp(-2N_e\alpha(\hat{p}-p)^2) \qquad \text{(A12)}$$

(Wright 1945; Robertson 1962; Rice 2004) where the constant C ensures that the probability density function integrates to one (*i.e.*, $C^{-1} = \int_0^1 [p(1-p)]^{-1}\exp(-2N_e\alpha(\hat{p}-p)^2)\,dp$).

Balancing selection effectively maintains polymorphism when the probability density near the equilibrium (p near $\hat{p}$) is high relative to the probability density near the boundaries of the distribution ($p = 1/2N_e$ and $p = 1 - 1/2N_e$). A measure of these relative densities (following Connallon & Clark, 2014) is given by:

$$R \approx \frac{1}{2N_e\hat{p}(1-\hat{p})(\exp(-2N_e\alpha\hat{p}^2) + \exp(-2N_e\alpha(1-\hat{p})^2))} \qquad \text{(A13)}$$

for which balancing selection is effective when R is large ($R \gg 1$), and drift dominates when R is small ($R < 1$). Using a cut-off of $R > 10$ to represent effectively strong balancing selection, eq. (13) implies the following minimum values of α ($\alpha_{\min}$) are required to ensure the stability of a balanced polymorphism:

$$\alpha_{\min} = \begin{cases} \dfrac{\log(20N_e\hat{p})}{2N_e\hat{p}^2} & \text{for } \hat{p} \le 0.5 \\ \dfrac{\log\big(20N_e(1-\hat{p})\big)}{2N_e(1-\hat{p})^2} & \text{for } \hat{p} \ge 0.5 \end{cases} \qquad \text{(A14)}$$

From eq. (A14), balancing selection should be effective at maintaining polymorphism when $\alpha > \alpha_{\min}$, whereas drift will impede maintenance when α falls substantially below this threshold.

Figure 2 illustrates conditions for effective balancing selection across the spectrum of polymorphic equilibrium frequency states ($0 < \hat{p} < 1$) and for three different effective population sizes (N_e = 1,000; N_e = 10,000; N_e = 100,000). Two special cases of moderately strong balancing selection (α = 0.01 and α = 0.05) in a small population (N_e = 1,000) illustrate how the probability density of the stationary distribution is concentrated near $p = \hat{p}$ when $\alpha > \alpha_{\min}$, whereas it accumulates near p = 0 when $\alpha < \alpha_{\min}$ (Fig. 2A, top versus bottom panels).

Appendix 3 - Linked variation

Long-term balanced polymorphisms can affect patterns of genetic diversity at linked and otherwise neutrally evolving loci, leading to characteristic signatures of inflated heterozygosity in genomic regions where the balanced polymorphism resides. With the exceptions of trans-species polymorphisms, it is these signatures that are used by current methods for the inference of balancing selection in genomic data (Bitarello et al., 2018; Cheng & DeGiorgio, 2020; Siewert & Voight, 2017). For a balanced polymorphism with alleles A_1 and A_2 that are stably maintained at equilibrium frequencies $\hat{p}$ and $1 - \hat{p}$ (respectively), the mean heterozygosity at a nearby neutral site is approximately:

$$\bar{H} = 4N_e\mu\left[1 + 2\hat{p}(1-\hat{p})\left(\frac{1}{2N_eR} + \frac{(1-2\hat{p})^2}{2 + 8N_eR\hat{p}(1-\hat{p})}\right)\right] \tag{A15}$$

where R is the total recombinational distance between the neutral site and the site of the balanced polymorphism, and μ is the mutation rate at the neutral site (the approximation, which assumes that $R >> \mu$, is obtained using mean coalescent times from p. 103 of Rice 2004).

The sum in the square brackets of eq. (A15) determines the degree of inflation of heterozygosity at the linked site relative to that of an unlinked locus, where the expected heterozygosity would be $\bar{H} = 4N\mu$. Recombination rate and the equilibrium frequency of the selected site also influence the width of the spike in neutral diversity that could be seen around a long-term balanced polymorphism. Let $R = nr$ represent the cumulative recombination rate between the selected site and a given neutral site, where n is the number of nucleotide sites separating the two and r is the recombination rate per site (*i.e.*, the probability of a recombination event between two directly adjacent nucleotide sites). Assuming that the polymorphism is intermediate ($\hat{p} = 0.5$), then the window around the selected site over which

neutral diversity is elevated by at least two-fold (*i.e.*: where $\frac{\bar{H}}{4N\mu} > 2$) will be $L = \frac{1}{2N_e r}$ nucleotides, which implies that species with large effective population sizes and large per-nucleotide recombination rates should exhibit particularly narrow windows of inflated heterozygosity around loci maintaining long-term balanced polymorphisms.
