# Supplementary material for: Characterizing the detectable and invisible fractions of genomic loci under balancing selection
Source: Mol Biol Evol. 2026 Oct 5;43(10):msag228. doi: 10.1093/molbev/msag228 (PMC13635458; doi:10.1093/molbev/msag228)
Supplement: Supplementary file 1 [file msag228_supplementary_data.zip › BrandtEtAL_SuppMat.pdf]

Supplementary Material — Characterising the detectable and invisible fractions of genomic loci under balancing selection

Débora Y. C. Brandt, Aida M. Andrés, Tim Connallon and Max Reuter

Contents

Supplementary Figures 5

Figure S1: Maintenance of polymorphism under overdominance 5

Figure S2: The effect of genetic drift on maintenance of polymorphism in simulations of overdominance 6

Figure S3: Maintenance of polymorphism under antagonistic pleiotropy 7

Figure S4: The effect of genetic drift on maintenance of polymorphism in simulations of antagonistic pleiotropy 8

Figure S5: Maintenance of polymorphism under sexually antagonistic selection 9

Figure S6: The effect of genetic drift on maintenance of polymorphism in simulations of sexually antagonistic selection 10

Figure S7: Power to detect balancing selection in simulations of overdominance using NCD 11

Figure S8: Power to detect balancing selection in simulations of antagonistic pleiotropy using NCD 11

Figure S9: Power to detect balancing selection in simulations of sexual antagonism using NCD 12

Figure S10: Power to detect balancing selection in simulations of overdominance using BalLeRMix 12

Figure S11: Power to detect balancing selection in simulations of antagonistic pleiotropy using BalLeRMix 13

Figure S12: Power to detect balancing selection in simulations of sexual antagonism using BalLeRMix 13

Figure S13: Comparison of power of NCD and BalLeRMix across all parameter combinations presented in the main text. 14

Figure S14: Power to detect balancing selection with NCD with a target frequency fixed to the theoretical equilibrium frequency 15

Figure S15: Power to detect balancing selection with NCD as a function of NCD window size 15

Figure S16: Fixed differences in our simulated data 16

Supplementary Methods 17

Supplementary Results 17

1. NCD with target frequency 0.5 19

1.1. Effects of equilibrium frequency, selection strength and selection mode in standard simulations 19

Table S1: Coefficients of a GLM for detection as a function of equilibrium frequency (pstar), strength of selection (selalpha), model of selection (seltype) and their interactions. 19

Table S2: Analysis of Deviance for the model shown in Table S1. AIC: 422.01. ... 20

1.2. Effects of recombination, mutation, selection onset and N_e in simulations of overdominance 21

Table S3: Analysis of Deviance for GLM including equilibrium frequency (pstar), selection strength (selalpha), recombination rate (rec_recoded, as $-\log_{10}(r)$) and their interactions as predictor variables. 21

Table S4: Analysis of Deviance for GLM including equilibrium frequency (pstar), selection strength (selalpha), mutation rate (mut_recoded, as $-\log_{10}(\mu)$) and their interactions as predictor variables. 22

Table S5: Analysis of Deviance for GLM including equilibrium frequency (pstar), selection strength (selalpha), allelic age (t_ne, in N_e generations) and their interactions as predictor variables. 23

Table S6: Analysis of Deviance for GLM including equilibrium frequency (pstar), selection strength (selalpha), N_e (NeK, in thousands) and their interactions as predictor variables. 24

2. NCD with target frequency set to the true equilibrium frequency 25

2.1 Effects of equilibrium frequency, selection strength and model of selection in reference simulations 25

Figure S17: Power of NCD as a function of equilibrium frequency ($\boldsymbol{p}$, panel A), selection strength ($\boldsymbol{\alpha}$, panel B), and model of selection (panel C). 25

Table S7: Coefficients of a GLM for detection as a function of equilibrium frequency (pstar), strength of selection (selalpha), model of selection (seltype) and their interactions. 26

Table S8: Analysis of Deviance for the model shown in Table S7. AIC: 444.34. ... 27

2.2. Effects of recombination, mutation, selection onset and N_e in simulations of overdominance 28

Figure S18: Power of NCD as a function of recombination rate (panel A), mutation rate (panel B), selection onset time (panel C) and effective population size (panel D). 28

Table S9: Analysis of Deviance for GLM including equilibrium frequency (pstar), selection strength (selalpha), recombination rate (rec_recoded, as $-\log_{10}(r)$) and their interactions as predictor variables. 29

Table S10: Analysis of Deviance for GLM including equilibrium frequency (pstar), selection strength (selalpha), mutation rate (mut_recoded, as $-\log_{10}(\mu)$) and their interactions as predictor variables. 30

Table S11: Analysis of Deviance for GLM including equilibrium frequency (pstar), selection strength (selalpha), allelic age (t_ne, in N_e generations) and their interactions as predictor variables. 31

Table S6: Analysis of Deviance for GLM including equilibrium frequency (pstar), selection strength (selalpha), N_e (NeK, in thousands) and their interactions as predictor variables. 32

3. BalLeRMix optimizing equilibrium frequency 33

3.1. Effects of equilibrium frequency, selection strength and model of selection in reference simulations 33

Figure S19: Power of BalLeRMix as a function of equilibrium frequency ($\boldsymbol{p}$, panel A), selection strength ($\boldsymbol{\alpha}$, panel B), and model of selection (panel C). 33

Table S13: Coefficients of a GLM for detection as a function of equilibrium frequency (pstar), strength of selection (selalpha), model of selection (seltype) and their interactions. 34

Table S14: Analysis of Deviance for the model shown in Table S13. AIC: 367.26. 35

3.2. Effects of recombination, mutation, selection onset and N_e in simulations of overdominance 36

Figure S20: Power of BalLeRMix as a function of recombination rate (panel A), mutation rate (panel B), selection onset time (panel C) and effective population size (panel D). 36

Table S15: Analysis of Deviance for GLM including equilibrium frequency (pstar), selection strength (selalpha), recombination rate (rec_recoded, as $-\log_{10}(r)$) and their interactions as predictor variables. 37

Table S16: Analysis of Deviance for GLM including equilibrium frequency (pstar), selection strength (selalpha), mutation rate (mut_recoded, as -$\log_{10}(\mu)$) and their interactions as predictor variables. 38

Table S17: Analysis of Deviance for GLM including equilibrium frequency (pstar), selection strength (selalpha), allelic age (t_ne, in N_e generations) and their interactions as predictor variables. 39

Table S18: Analysis of Deviance for GLM including equilibrium frequency (pstar), selection strength (selalpha), N_e (NeK, in thousands) and their interactions as predictor variables. 40

4. BalLeRMix setting equilibrium frequency to the theoretical value 41

4.1 Effects of equilibrium frequency, selection strength and model of selection in reference simulations 41

Figure S21: Power of BalLeRMix as a function of equilibrium frequency ($\boldsymbol{p}$, panel A), selection strength ($\boldsymbol{\alpha}$, panel B), and model of selection (panel C). 41

Table S19: Coefficients of a GLM for detection as a function of equilibrium frequency (pstar), strength of selection (selalpha), model of selection (seltype) and their interactions. 42

Table S20: Analysis of Deviance for the model shown in Table S19. AIC: 450.7. 43

4.2. Effects of recombination, mutation, selection onset and N_e in simulations of overdominance 44

Figure S22: Power of BalLeRMix as a function of recombination rate (panel A), mutation rate (panel B), selection onset time (panel C) and effective population size (panel D). 44

Table S21: Analysis of Deviance for GLM including equilibrium frequency (pstar), selection strength (selalpha), recombination rate (rec_recoded, as -$\log_{10}(r)$) and their interactions as predictor variables. 45

Table S22: Analysis of Deviance for GLM including equilibrium frequency (pstar), selection strength (selalpha), mutation rate (mut_recoded, as -$\log_{10}(\mu)$) and their interactions as predictor variables. 46

Table S23: Analysis of Deviance for GLM including equilibrium frequency (pstar), selection strength (selalpha), allelic age (t_ne, in N_e generations) and their interactions as predictor variables. 47

Table S24: Analysis of Deviance for GLM including equilibrium frequency (pstar), selection strength (selalpha), N_e (NeK, in thousands) and their interactions as predictor variables. 48

Supplementary Figures

Figure S1: Maintenance of polymorphism under overdominance

Maintenance of polymorphism is 100% in our reference simulations (see Table 3 for parameter values). Maintenance is recorded out of 100 initiated simulations with balancing selection. Panel A shows the grid of weak selection coefficients; panel B shows the grid of strong selection coefficients.

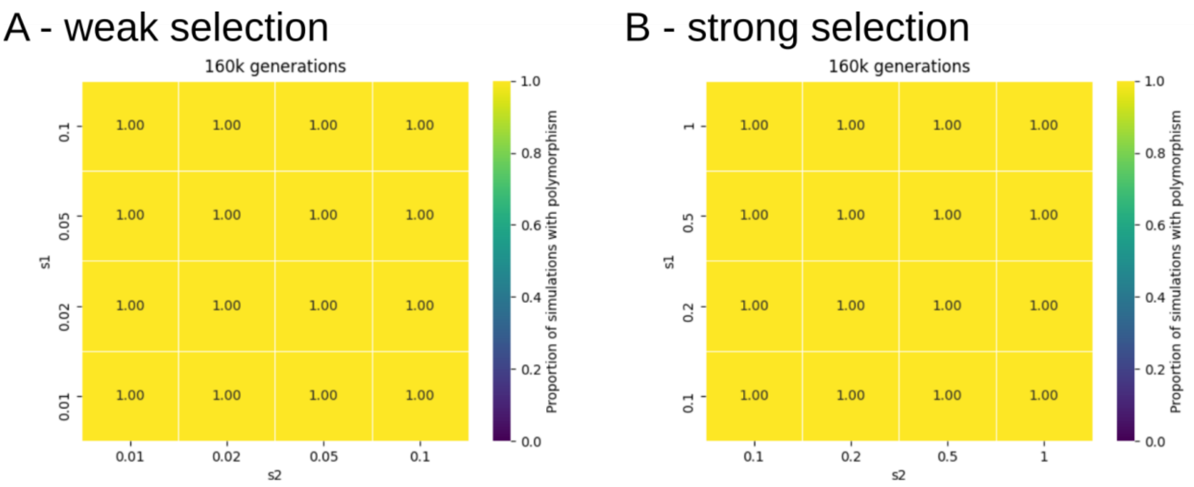

Figure S2: The effect of genetic drift on maintenance of polymorphism in simulations of overdominance

Maintenance of polymorphism is reduced with increased genetic drift (i.e. lower effective population size (N_e), panels C and D, compared to higher N_e in panels A and B). This is particularly true at the edges of the parameter space, where selection coefficients are most asymmetrical and equilibrium frequencies closest to 0 and 1. Panels B and D show maintenance at T=16N_e generations (twice as long as in the standard simulations, T=8N_e generations). Maintenance is recorded out of 100 initiated simulations with balancing selection. Simulations were run with recombination and mutation rates at 10^{-8}.

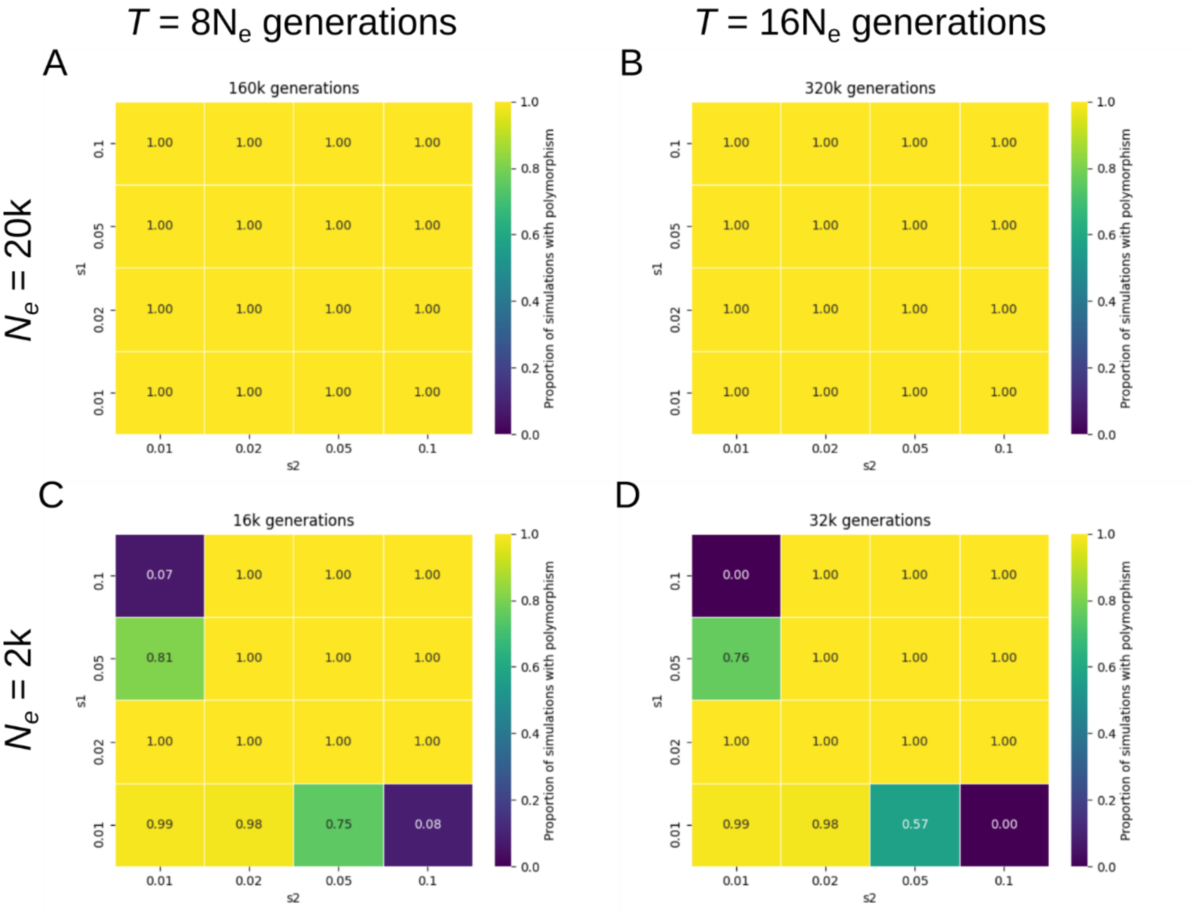

Figure S3: Maintenance of polymorphism under antagonistic pleiotropy

As predicted by theory (Fig. 1) polymorphisms under antagonistic pleiotropy are preferentially maintained when selection is strong and symmetrical. Maintenance increases with dominance reversal (*h*=0.25, panels B and D, compared to codominance, *h*=0.5), panels A and C, and with stronger selection coefficients (panels C and D). Maintenance is recorded out of 100 initiated reference simulations with balancing selection (see Table 4 for parameter values of reference simulations).

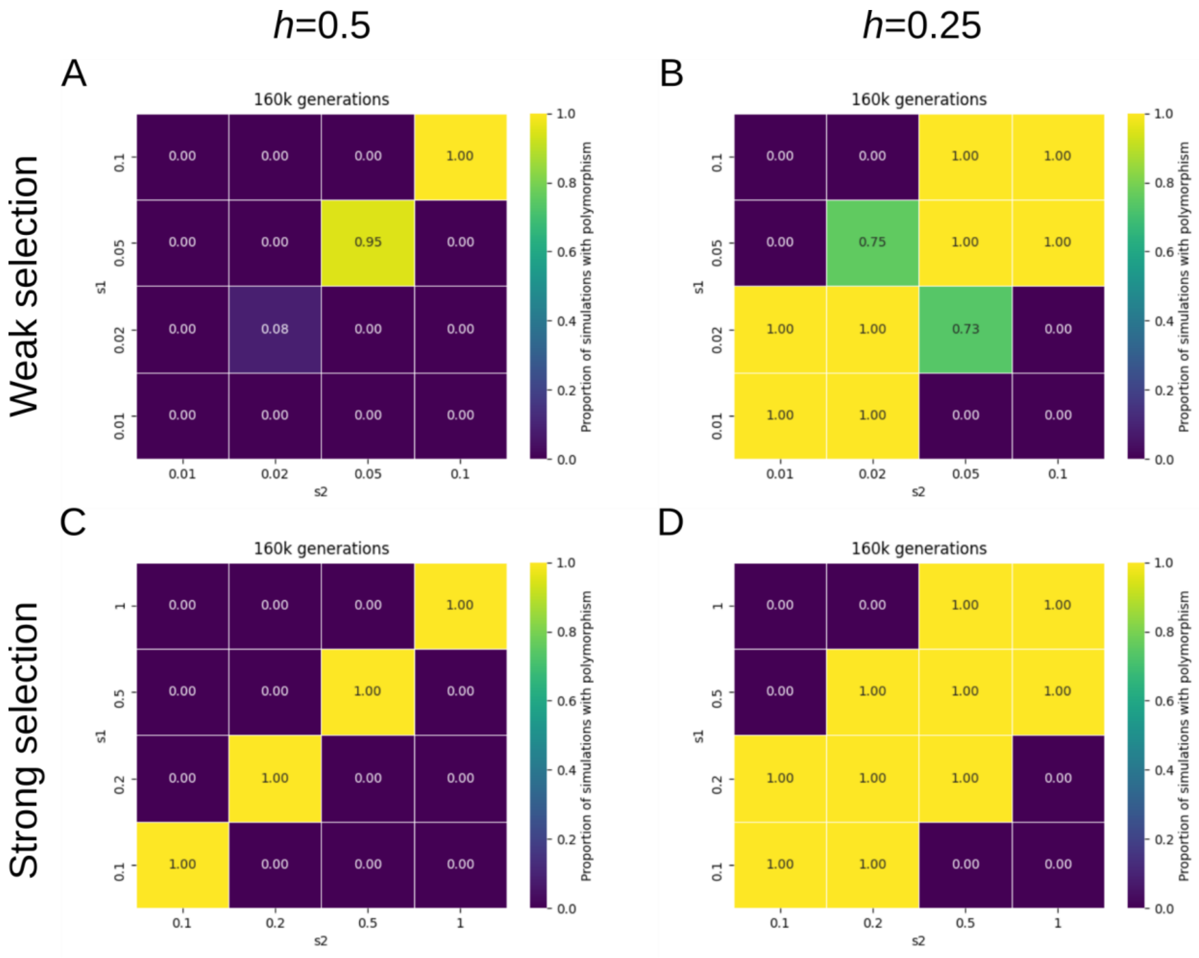

Figure S4: The effect of genetic drift on maintenance of polymorphism in simulations of antagonistic pleiotropy

Maintenance of polymorphism is reduced with increased genetic drift (i.e. lower effective population size (N_e), panels A and B, compared to higher N_e in Figures S3A and B). The effect of drift is particularly strong under conditions where selection is weaker, i.e. towards the edges of the parameter space where selection coefficients are more asymmetrical. Maintenance is recorded out of 100 initiated simulations with balancing selection. Simulations were run for $8N_e$=16k generations. See Table 4 for parameter values not shown in the figure.

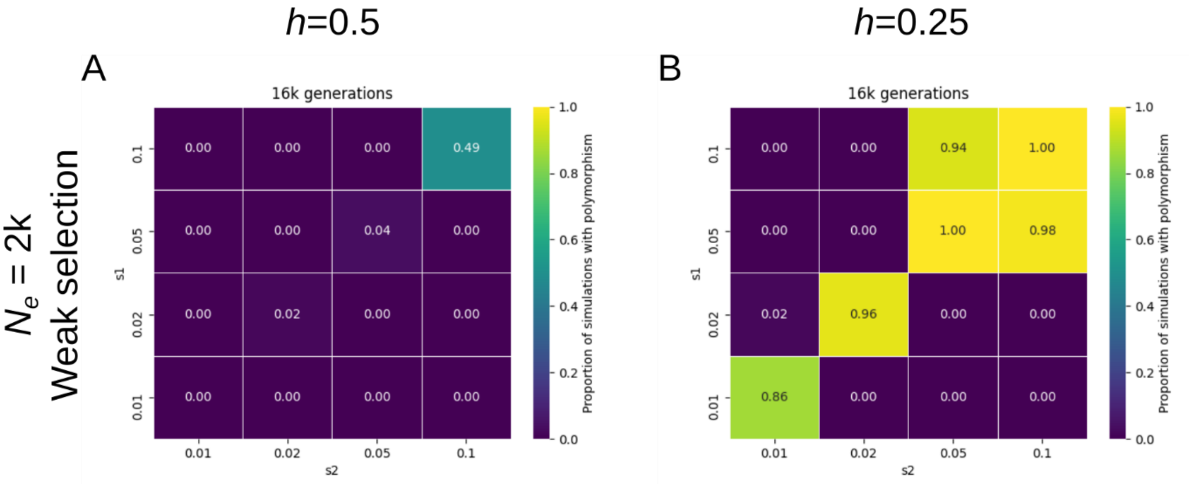

Figure S5: Maintenance of polymorphism under sexually antagonistic selection

As predicted by theory (Fig. 1) polymorphisms under sexually antagonistic selection are preferentially maintained when selection is strong and symmetrical. Maintenance increases with dominance reversal (*h*=0.25, panels B and D, compared to codominance, *h*=0.5, panels A and C), and with stronger selection coefficients (panels C and D). Maintenance is recorded out of 100 initiated reference simulations with balancing selection (see Table 4 for parameter values of reference simulations).

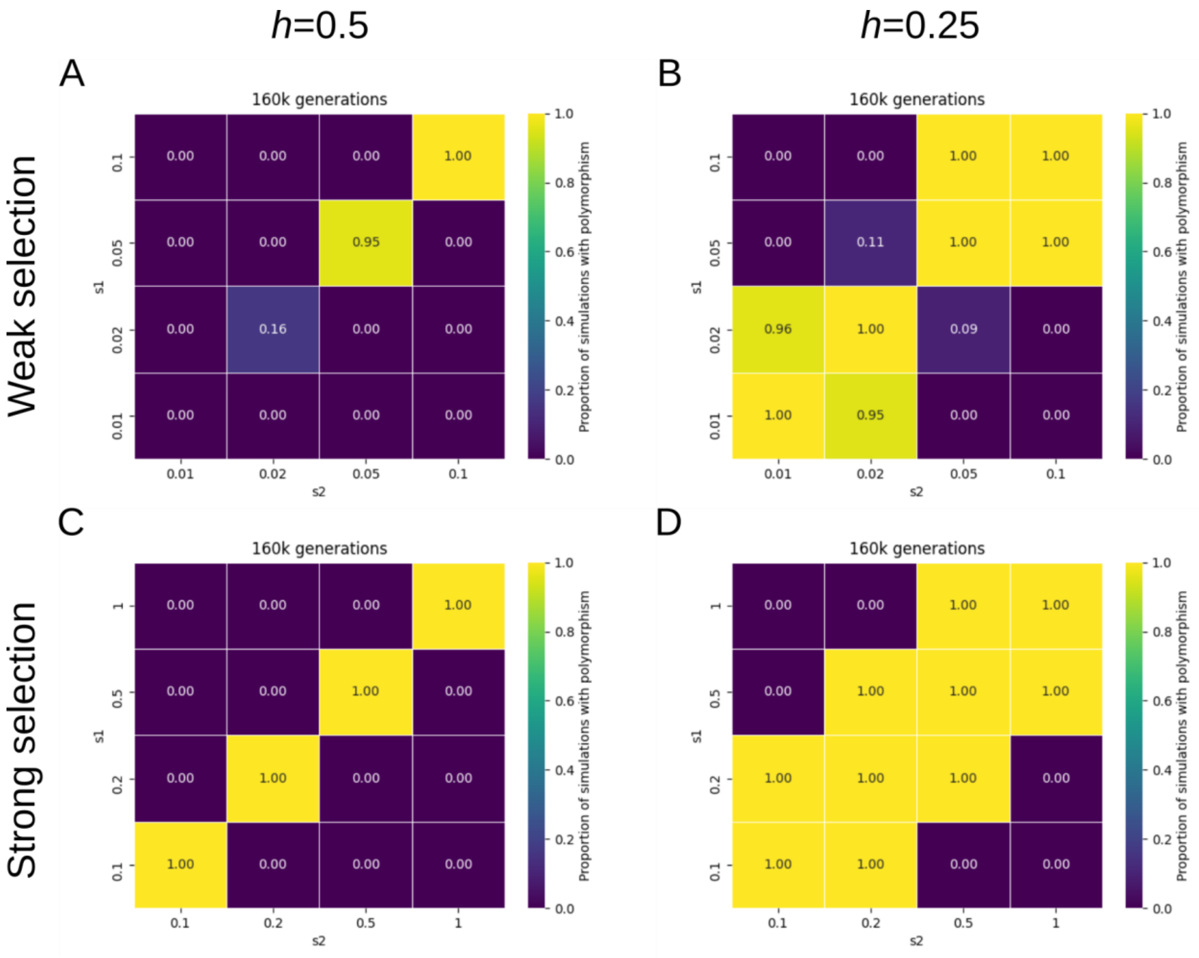

Figure S6: The effect of genetic drift on maintenance of polymorphism in simulations of sexually antagonistic selection

Maintenance of polymorphism is reduced with increased genetic drift (i.e. lower effective population size (N_e), panels A and B, compared to higher N_e in Figures S5A and B). The effect of drift is particularly strong under conditions where selection is weaker, i.e. towards the edges of the parameter space where selection coefficients are more asymmetrical. Maintenance is recorded out of 100 initiated simulations with balancing selection. Simulations were run for $8N_e$=16k generations. See Table 4 for parameter values not shown in the figure.

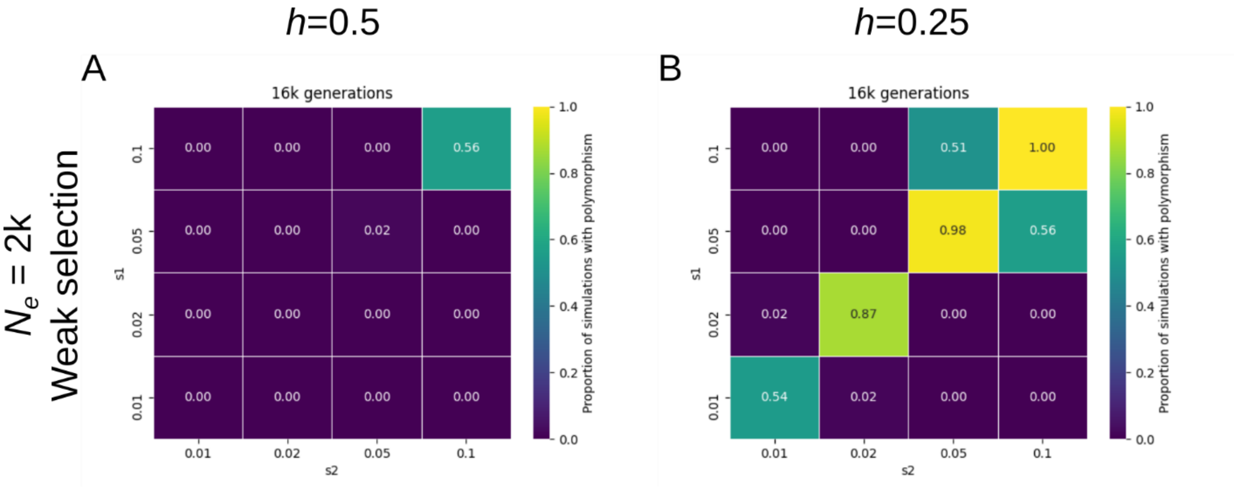

Figure S7: Power to detect balancing selection in simulations of overdominance using NCD

Power is shown for reference simulations (see Table 4 for parameter values).

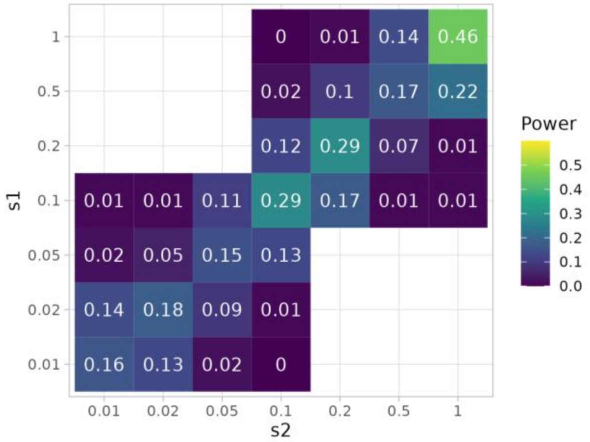

Figure S8: Power to detect balancing selection in simulations of antagonistic pleiotropy using NCD

Power is shown for reference simulations (see Table 4 for parameter values). A) Simulations under codominance (*h*=0.5); B) Simulations under dominance reversal (*h*=0.25).

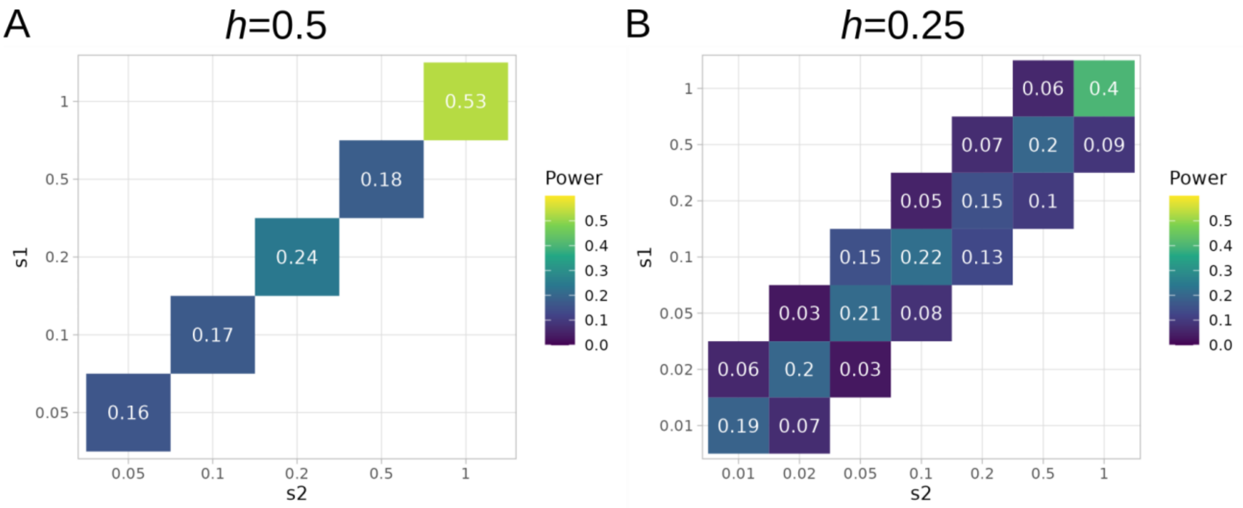

Figure S9: Power to detect balancing selection in simulations of sexual antagonism using NCD

Power is shown for reference simulations (see Table 4 for parameter values). A) Simulations under codominance (*h*=0.5); B) Simulations under dominance reversal (*h*=0.25).

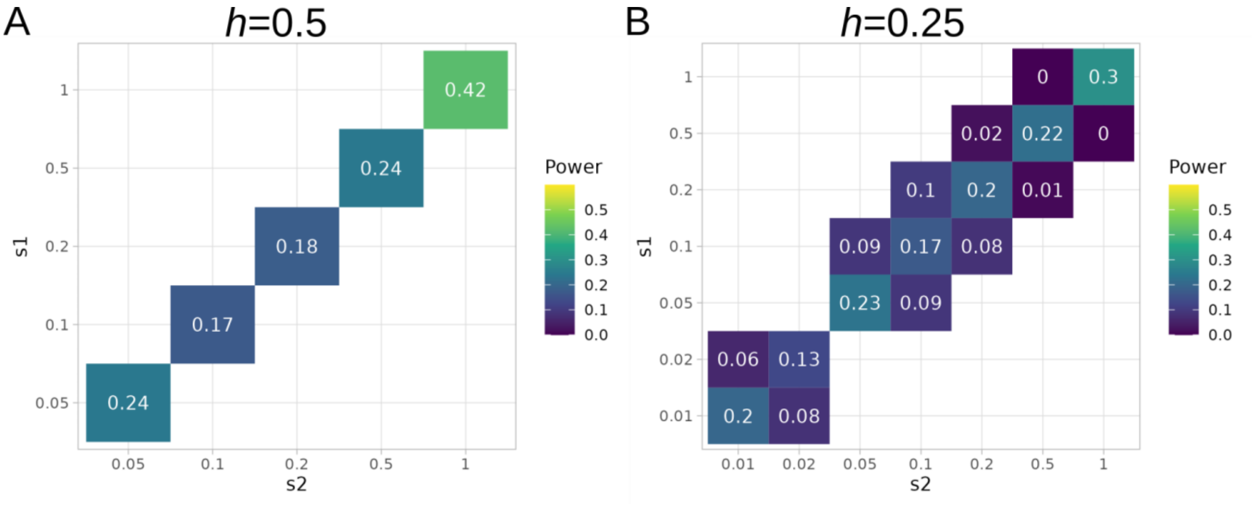

Figure S10: Power to detect balancing selection in simulations of overdominance using BalLeRMix

Power is shown for reference simulations (see Table 4 for parameter values), and running BalLeRMix allowing it to optimise equilibrium frequencies.

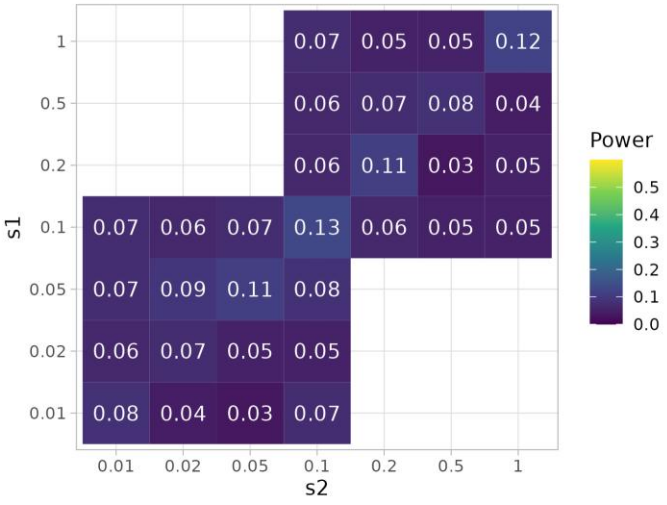

Figure S11: Power to detect balancing selection in simulations of antagonistic pleiotropy using BalLeRMix

Power is shown for reference simulations (see Table 4 for parameter values). A) Simulations under codominance (*h*=0.5); B) Simulations under dominance reversal (*h*=0.25).

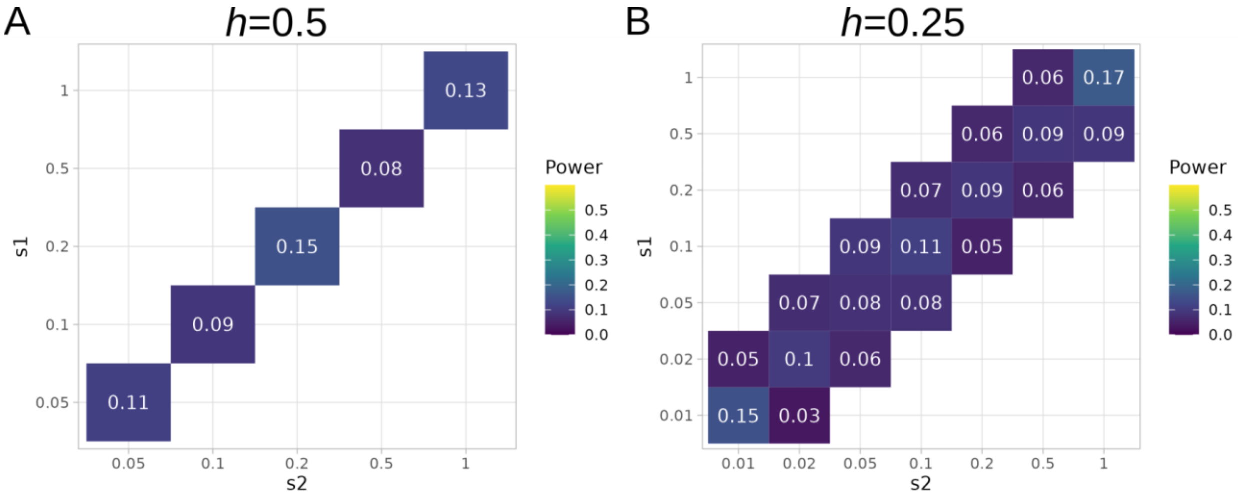

Figure S12: Power to detect balancing selection in simulations of sexual antagonism using BalLeRMix

Power is shown for reference simulations (see Table 4 for parameter values). A) Simulations under codominance (*h*=0.5); B) Simulations under dominance reversal (*h*=0.25).

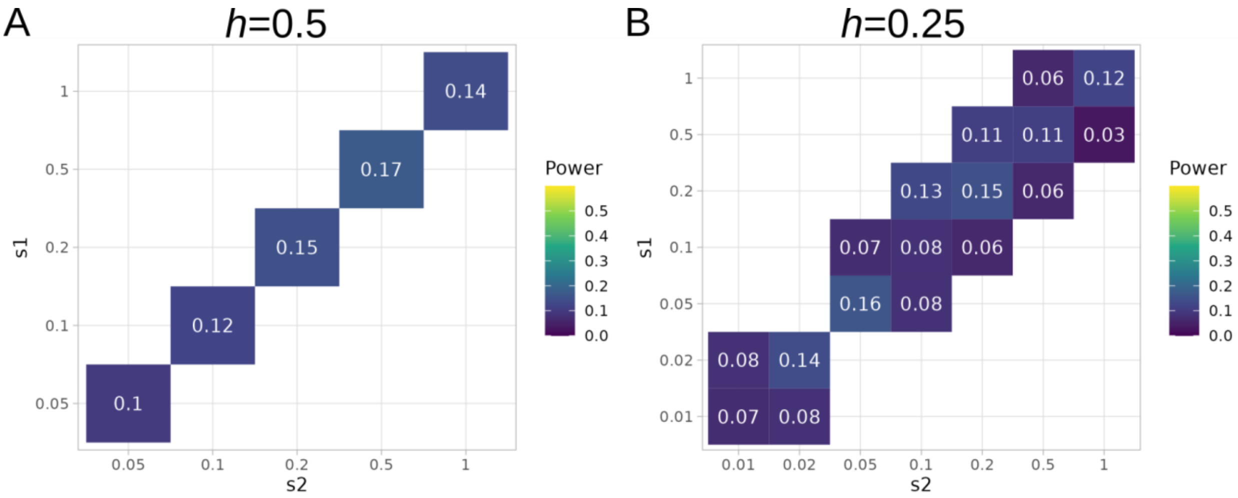

Figure S13: Comparison of power of NCD and BalLeRMix across all parameter combinations presented in the main text.

Comparisons are shown for a scenario where the equilibrium is not known and the target frequency set to tf=0.5 for NCD and the equilibrium frequency optimised for BalLeRMix (panel A), and a scenario where the equilibrium/target frequencies are set to the known theoretical equilibrium (panel B). Parameters for reference simulations ("Ref") are as in Table 4. Other simulations have a single parameter changed at a time, relative to reference simulations: "-Ne" has effective population size of 2,000; "+mut" has a mutation rate of 10^{-7}; "+rec" has a recombination rate of 10^{-7}; "+t" has selection onset time $16N_e$ generations ago. Pearson correlation coefficient and associated p-value are shown on the plots. The diagonal line shows equal power, a line with an intercept of 0 and a slope of 1.

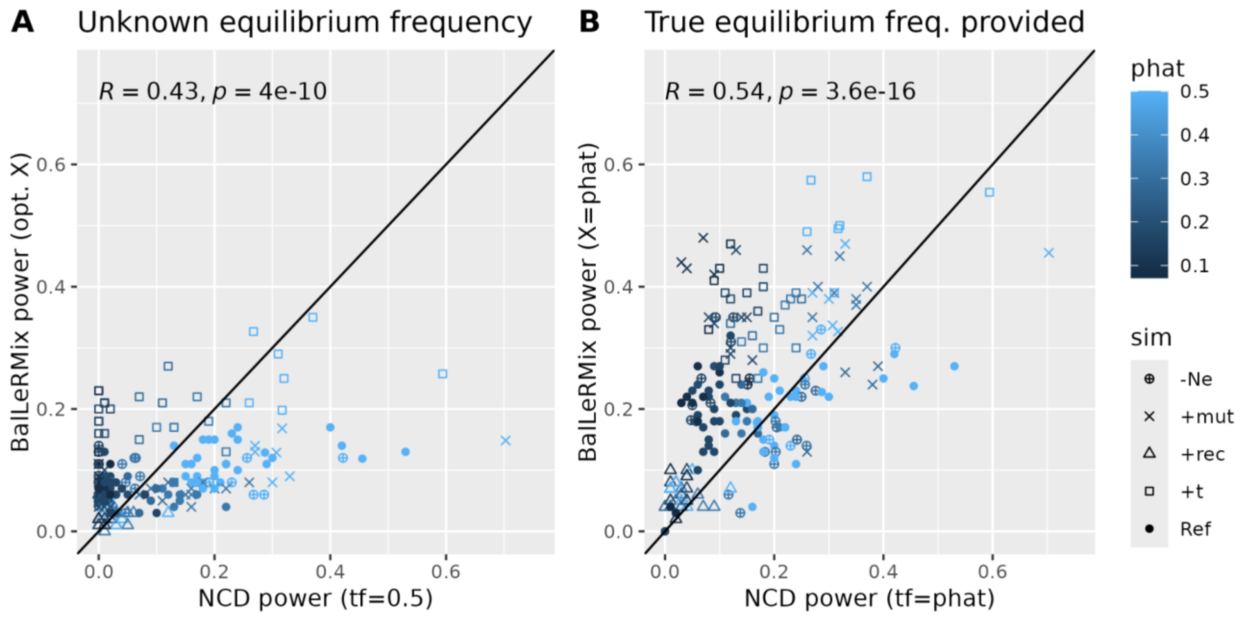

Figure S14: Power to detect balancing selection with NCD with a target frequency fixed to the theoretical equilibrium frequency

Power is shown for simulations under overdominance and with parameter values from the reference set (Table 4).

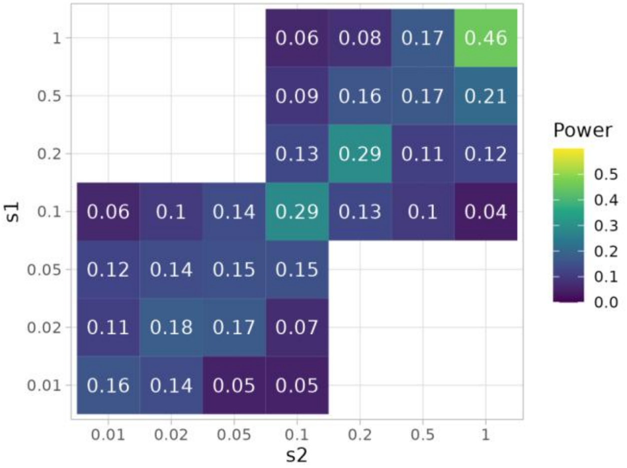

Figure S15: Power to detect balancing selection with NCD as a function of NCD window size

The relationship between power and window size is shown for reference simulations (panel A) and simulations with elevated recombination rate (r=10^{-7}, panel B). Power was computed from data simulated under overdominance. The color legend shows the equilibrium frequency ($\hat{p}$). Datapoints that are missing at smaller window sizes are due to small windows not having enough informative sites to compute NCD. A) Reference simulations. B) Simulations with increased recombination rate.

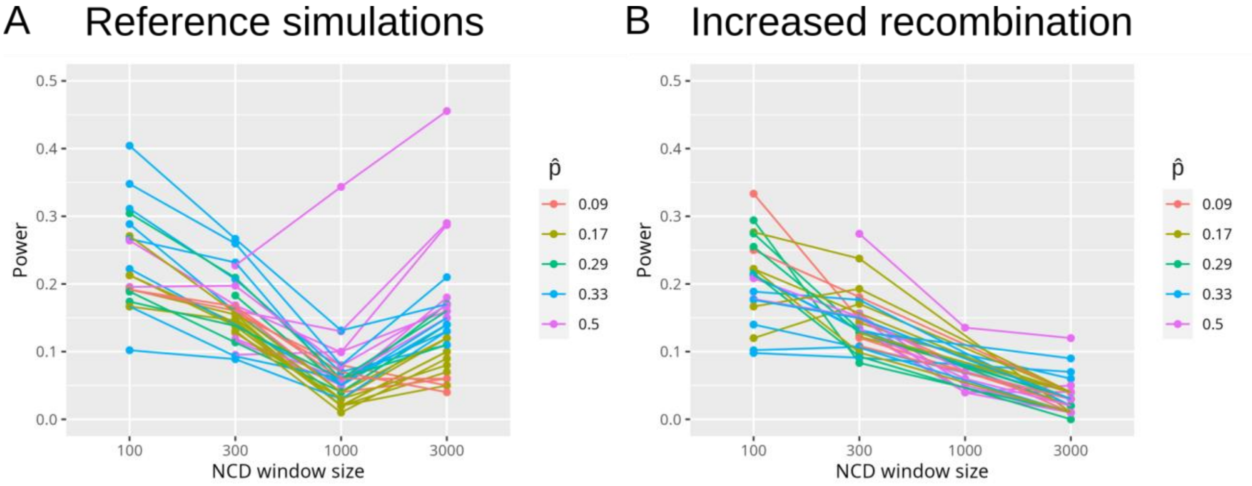

Figure S16: Fixed differences in our simulated data

Fixed differences are mutations that accumulate in the branch connecting an outgroup to the most recent common ancestor of the ingroup (red branches). The fixed differences that are informative for the detection of balancing selection are those that accumulate in neutral regions over the excess time during which balanced polymorphisms segregate in a population (solid red branch). In contrast, older fixed differences or those that occur in the branch leading to the outgroup accumulate at the same rate under balancing selection or neutrality and thus are uninformative (dashed red branches). We used a combination of forward and backwards simulations that allowed us to include most of the informative fixed differences. Forward simulations with balancing selection (left tree) could never fully coalesce. This was by design, since we started these simulations with a single-copy mutation present at the selected site and conditioned on this site still being polymorphic among sampled individuals at the end of the simulation. Full coalescence was then achieved via neutral backward simulations (recapitation, dashed blue branches). In contrast, coalescence very frequently occurred in the forward phase of the neutral simulations (right tree), resulting in a stem branch (solid red branch underlaid in light red) along which fixed differences could arise within the simulated ingroup. A subset of informative fixed differences is not included in our approach: those that could have occurred under neutrality in the time taken for simulations with selection to coalesce during recapitation (shaded gray area). On average, we simulate 68% and 87% of the informative fixed differences in forward simulations of $8N_e$ and $16N_e$ generations, respectively.

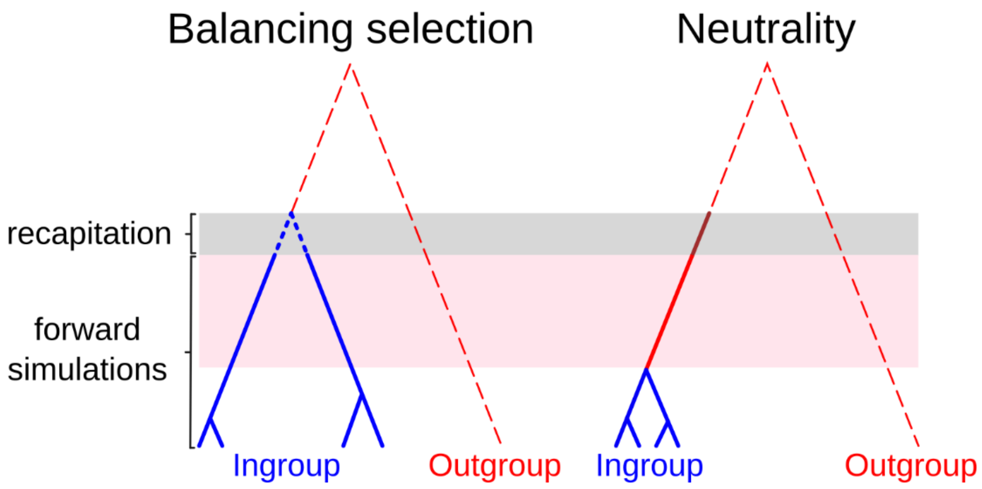

Supplementary Methods

In addition to our main analyses using the NCD (non-central deviation) method (Bitarello et al., 2018) to detect balancing selection, we here present complementary results using the software BalLeRMix, version 2.5 (Cheng & DeGiorgio, 2020). Similarly to NCD2, we used the B2 statistic from BalLeRMix, which also considers fixed differences in its likelihood calculations.

First, we ran 3000 replicates of neutral simulations for each combination of effective population size (N_e), recombination rate, mutation rate and selection onset time, to compose the BalLeRMix helper file with the background frequency spectrum. VCF files from the simulations were converted to BalLeRMix input format (INFILE1) using a custom script and the BalLeRMix helper file was generated using the following command:

```
python3 BalLeRMix/software/BalLeRMix_v2.5.py -i $INFILE1 --
getSpect --spect $SPECT
```

Next, we calculated the B2 statistic for each simulated sequence of 10kb using the command

```
python3 BalLeRMix/software/BalLeRMix_v2.5.py -i $INFILE2 -o
$OUTFILE.B2 --spect $SPECT --physPos --rec $RECcM
```

Where $RECcM is the recombination rate used in the simulations, converted to centi-Morgans per nucleotide (*i.e.* multiplied by 100).

As described for NCD2, we assessed the statistical significance of the B2 values by generating a null distribution by running 1000 neutral simulations for each combination of relevant parameters (N_e, μ and r) and calculating B2 for each. We then calculated a P-value for a B2 value from a simulation with selection by determining the proportion of B2 values from the null distribution (with corresponding N_e, μ and r) that was lower than the B2 value with selection. Power was then calculated as the proportion of the 100 simulations generated under a particular set of parameters for which $P \leq 0.01$.

Supplementary Results

Here, we present the full details of the power analyses presented in the main text, as well as complementary analyses. In the first section, we show the full results of generalised linear models (GLMs) used to test the effect of various predictors on the detection of balanced loci using the NCD method with a target frequency fixed at 0.5,

the results of which were in Tables 2 and 3 of the main text. In section 2, we present results of equivalent analyses for the case where balancing selection is detected with NCD using a target frequency equal to the theoretical equilibrium frequency of the balanced polymorphism to be detected. This is a hypothetical best-case scenario that allows us to evaluate variation in power that arises due to the equilibrium frequency alone (section 2), compared to variation that in addition arises due to a mismatch between the NCD target frequency and the true equilibrium frequency (section 1, main text). In sections 3 and 4 we replicate the same analyses while using an additional method to detect balancing selection, BalLeRMix. We use BalLeRMix as a representative of a likelihood-based test for balancing selection (as opposed to NCD, which is a summary statistic). As with NCD, we show results based on BalLeRMix run under two modes: one where the program optimises the equilibrium frequency from the data (the recommended usage, representative of how they would be applied in real data analyses, section 3), and one where the true equilibrium frequency is provided to the program (flag -fixX, section 4).

With NCD, equilibrium frequency is the strongest predictor of power (Tables S2 and S8). The same is true for BalLeRMix when we allowed it to optimize the equilibrium frequency (Table S14). When BalLeRMix is provided with the true equilibrium frequency (Table S20), the effect of equilibrium frequency is only present in interactions with the model of selection and with selection strength (which are also present in NCD).

Selection strength is the second strongest predictor of NCD power (Tables S2 and S8). It also has a significant effect on BalLeRMix power when the equilibrium frequency is set to the true value (Table S20), but not when the approach is allowed to optimize the equilibrium frequency (Table S14).

The model of selection only affects NCD power via interactions with equilibrium frequency and selection strength (Tables S2 and S8), but has a significant effect on power with BalLeRMix (Tables S14, S20).

The negative effect of recombination rate on power to detect balancing selection is highly consistent among all four approaches we explored (see odds ratios (OR) of 0.311, 0.431, 0.56, 0.213 for recombination in Tables S3, S9, S15 and S21). The positive effect of mutation rate and allelic age is also consistent among the four approaches we tested (OR of 1.016, 2.407, 1.395, 1.772 for mutation rate in Tables S4, S10, S16, S22 and OR of 1.085, 1.034, 1.22, 1.044 for allelic age in Tables S5, S11, S17, S23). The effective population size has weaker and less consistent effects on power across methods (Tables S6, S12, S18, S24).

1. NCD with target frequency 0.5

1.1. Effects of equilibrium frequency, selection strength and selection mode in standard simulations

Table S1: Coefficients of a GLM for detection as a function of equilibrium frequency (pstar), strength of selection (selalpha), model of selection (seltype) and their interactions.

Significance codes: 0 ‘***’ 0.001 ‘**’ 0.01 ‘*’ 0.05 ‘.’ 0.1 ‘ ’ 1.

	Estimate	Std. Error	z value	Pr(>|z|)	Significance
(Intercept)	-4.4875	0.2931	-15.309	< 2e-16	***
pstar	6.3548	0.7022	9.05	< 2e-16	***
selalpha	-0.2468	0.3957	-0.624	0.532815	
seltype_recodedAP	-1.7024	0.6437	-2.645	0.008178	**
seltype_recodedSA	-0.6502	0.5155	-1.261	0.207165	
pstar:selalpha	1.5922	0.8957	1.778	0.075461	.
pstar:seltype_recodedAP	3.1495	1.3648	2.308	0.02102	*
pstar:seltype_recodedSA	0.9753	1.1285	0.864	0.387436	
selalpha:seltype_recodedAP	1.2914	1.2403	1.041	0.297778	
selalpha:seltype_recodedSA	4.9126	1.4564	3.373	0.000743	***
pstar:selalpha:seltype_recodedAP	-1.5145	2.5765	-0.588	0.556646	
pstar:selalpha:seltype_recodedSA	-8.0104	3.0772	-2.603	0.009238	**

Table S2: Analysis of Deviance for the model shown in Table S1. AIC: 422.01.
Significance codes: 0 ‘***’ 0.001 ‘**’ 0.01 ‘*’ 0.05 ‘.’ 0.1 ‘ ’ 1.

	Df	Deviance	Resid. Df	Resid. Dev	Pr(>Chi)	Significance
NULL			76	1029.52		
pstar	1	781.59	75	247.94	< 2.2e-16	***
selalpha	1	66.37	74	181.57	3.74E-16	***
seltype_recoded	2	1.31	72	180.26	0.5185712	
pstar:selalpha	1	4.11	71	176.15	0.0427196	*
pstar:seltype_recoded	2	14.15	69	162	0.0008478	***
selalpha:seltype_recoded	2	19.58	67	142.42	5.59E-05	***
pstar:selalpha:seltype_recoded	2	6.85	65	135.57	0.0326275	*

1.2. Effects of recombination, mutation, selection onset and N_e in simulations of overdominance

Tables below show full results for the GLMs summarised in Table 3.

Table S3: Analysis of Deviance for GLM including equilibrium frequency (pstar), selection strength (selalpha), **recombination rate** (rec_recoded, as -$\log_{10}(r)$) and their interactions as predictor variables.

The table provides information from the Analysis of Deviance (degrees of freedom–Df, Deviance and their residuals, P-value, percentage of deviance explained–Perc Dev), the estimated slope (b) and the odds ratio (OR=exp(b)) for main effects. Predictors that include recombination rate, have a significant effect (p<0.05), and percentage of deviance explained higher than 1% are highlighted.

	Df	Deviance	Resid. Df	Resid. Dev	Pr(>Chi)	Perc Dev	b	OR
NULL	NA	NA	61	637.8	NA	NA	-13.8	0
pstar	1	282.5	60	355.3	0.000	44.3	-9.5	0
selalpha	1	28.0	59	327.3	0.000	4.4	5.0	147.4
**rec_recoded**	**1**	**215.8**	**58**	**111.5**	**0.000**	**33.8**	**-1.2**	**0.301**
pstar:selalpha	1	2.8	57	108.6	0.091	0.5	-7.0	-
pstar:rec_recoded	1	5.2	56	103.5	0.023	0.8	-2.0	-
selalpha:rec_recoded	1	0.9	55	102.6	0.342	0.1	0.7	-
pstar:selalpha:rec_recoded	1	0.3	54	102.2	0.562	0.1	-1.1	-

Table S4: Analysis of Deviance for GLM including equilibrium frequency (pstar), selection strength (selalpha), **mutation rate** (mut_recoded, as $-\log_{10}(\mu)$) and their interactions as predictor variables.

The table provides information from the Analysis of Deviance (degrees of freedom–Df, Deviance and their residuals, P-value, percentage of deviance explained–Perc Dev), the estimated slope (b) and the odds ratio (OR=exp(b)) for main effects. Predictors that include mutation rate, have a significant effect (p<0.05), and percentage of deviance explained higher than 1% are highlighted.

	Df	Deviance	Resid. Df	Resid. Dev	Pr(>Chi)	Perc Dev	b	OR
NULL	NA	NA	61	988.6	NA	NA	-4.4	0.013
pstar	1	735.9	60	252.7	0.000	74.44	13.4	629610.38
selalpha	1	53.6	59	199.1	0.000	5.42	-5.1	0.006
**mut_recoded**	**1**	**22.8**	**58**	**176.3**	**0.000**	**2.31**	**0.0**	**1.016**
pstar:selalpha	1	15.0	57	161.3	0.000	1.52	14.9	-
pstar:mut_recoded	1	6.5	56	154.8	0.011	0.66	0.9	-
selalpha:mut_recoded	1	0.7	55	154.1	0.408	0.07	-0.6	-
pstar:selalpha:mut_recoded	1	1.7	54	152.4	0.192	0.17	1.7	-

Table S5: Analysis of Deviance for GLM including equilibrium frequency (pstar), selection strength (selalpha), **allelic age** (t_ne, in N_e generations) and their interactions as predictor variables.

The table provides information from the Analysis of Deviance (degrees of freedom–Df, Deviance and their residuals, P-value, percentage of deviance explained–Perc Dev), the estimated slope (b) and the odds ratio (OR=exp(b)) for main effects. Predictors that include allelic age, have a significant effect (p<0.05), and percentage of deviance explained higher than 1% are highlighted.

	Df	Deviance	Resid. Df	Resid. Dev	Pr(>Chi)	Perc Dev	b	OR
NULL	NA	NA	123	1734.2	NA	NA	-5.9	0.003
pstar	1	796.2	122	938.0	0.000	45.9	5.3	191.582
selalpha	1	66.4	121	871.6	0.000	3.8	-0.1	0.924
**t_ne**	**1**	**508.9**	**120**	**362.7**	**0.000**	**29.3**	**0.1**	**1.085**
pstar:selalpha	1	12.2	119	350.5	0.000	0.7	1.8	-
pstar:t_ne	1	9.7	118	340.8	0.002	0.6	0.2	-
selalpha:t_ne	1	4.1	117	336.7	0.044	0.2	0.0	-
pstar:selalpha:t_ne	1	0.1	116	336.7	0.819	0.0	0.0	-

Table S6: Analysis of Deviance for GLM including equilibrium frequency (pstar), selection strength (selalpha), N_e (NeK, in thousands) and their interactions as predictor variables.

The table provides information from the Analysis of Deviance (degrees of freedom–Df, Deviance and their residuals, P-value, percentage of deviance explained–Perc Dev), the estimated slope (b) and the odds ratio (OR=exp(b)) for main effects. Predictors that include N_e, have a significant effect (p<0.05), and percentage of deviance explained higher than 1% are highlighted.

	Df	Deviance	Resid. Df	Resid. Dev	Pr(>Chi)	PercDev	b	OR
NULL	NA	NA	59	738.5	NA	NA	-7.5	0.001
pstar	1	575.3	58	163.2	0.000	77.9	12.8	353141.89
selalpha	1	25.7	57	137.4	0.000	3.5	-0.7	0.483
**NeK**	**1**	**15.2**	**56**	**122.3**	**0.000**	**2.1**	**0.2**	**1.165**
pstar:selalpha	1	4.0	55	118.3	0.047	0.5	2.2	-
**pstar:NeK**	**1**	**35.8**	**54**	**82.5**	**0.000**	**4.9**	**-0.3**	**-**
selalpha:NeK	1	1.2	53	81.3	0.274	0.2	0.0	-
pstar:selalpha:NeK	1	0.1	52	81.3	0.797	0.0	0.0	-

2. NCD with target frequency set to the true equilibrium frequency

2.1 Effects of equilibrium frequency, selection strength and model of selection in reference simulations

Figure S17: Power of NCD as a function of equilibrium frequency ($\hat{p}$, panel A), selection strength (α, panel B), and model of selection (panel C).

Results shown are for reference simulations, and NCD target frequency set to the true equilibrium frequency. On panels A and C points were horizontally jittered for visualization purposes. The three panels show the same data points, with power on the vertical axis plotted against different horizontal axes of variation.

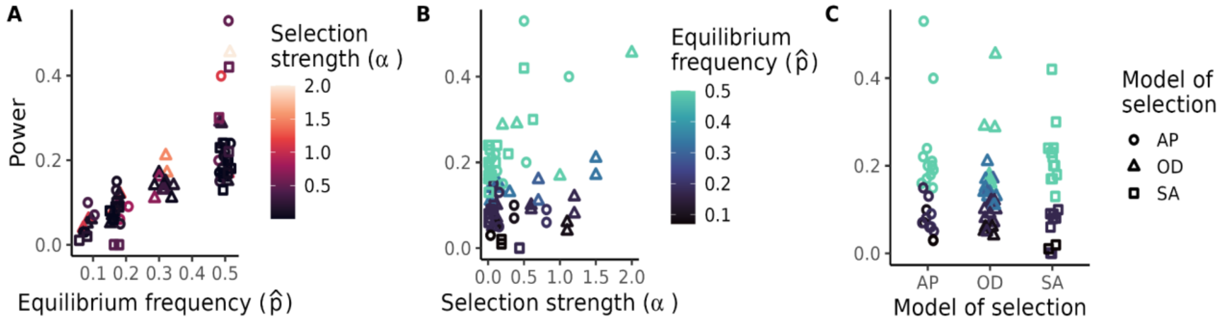

Table S7: Coefficients of a GLM for detection as a function of equilibrium frequency (pstar), strength of selection (selalpha), model of selection (seltype) and their interactions.

Significance codes: 0 ‘***’ 0.001 ‘**’ 0.01 ‘*’ 0.05 ‘.’ 0.1 ‘ ’ 1.

	Estimate	Std. Errod	z value	Pr(>|z|)	Significance
(Intercept)	-2.8126	0.204	-13.785	< 2e-16	***
pstar	2.7368	0.5421	5.048	4.46E-07	***
selalpha	-0.2507	0.2742	-0.914	0.3606	
seltype_recodedAP	-0.1271	0.2901	-0.438	0.6613	
seltype_recodedSA	0.3268	0.3421	0.955	0.3395	
pstar:selalpha	1.6191	0.6683	2.423	0.0154	*
pstar:seltype_recodedAP	0.2804	0.719	0.39	0.6965	
pstar:seltype_recodedSA	-0.7112	0.813	-0.875	0.3817	
selalpha:seltype_recodedAP	-0.2381	0.6359	-0.374	0.708	
selalpha:seltype_recodedSA	-11.419	2.8646	-3.986	6.71E-05	***
pstar:selalpha:seltype_recodedAP	1.4801	1.4356	1.031	0.3026	
pstar:selalpha:seltype_recodedSA	24.6335	5.8113	4.239	2.25E-05	***

Table S8: Analysis of Deviance for the model shown in Table S7. AIC: 444.34.

Significance codes: 0 ‘***’ 0.001 ‘**’ 0.01 ‘*’ 0.05 ‘.’ 0.1 ‘ ’ 1.

	Df	Deviance	Resid. Df	Resid. Dev	Pr(>Chi)	Significance
NULL			76	582.77		
pstar	1	362.96	75	219.82	< 2.2e-16	***
selalpha	1	47.66	74	172.15	5.06E-12	***
seltype_recoded	2	1.71	72	170.44	0.424536	
pstar:selalpha	1	13.64	71	156.8	0.000221	***
pstar:seltype_recoded	2	13.68	69	143.12	0.001069	**
selalpha:seltype_recoded	2	3.9	67	139.22	0.142380	
pstar:selalpha:seltype_recoded	2	33.38	65	105.83	5.64E-08	***

2.2. Effects of recombination, mutation, selection onset and N_e in simulations of overdominance

Figure S18: **Power of NCD as a function of recombination rate (panel A), mutation rate (panel B), selection onset time (panel C) and effective population size (panel D).**

Power to detect balancing selection in our reference set of simulations (points on the left of panels A-C and on the right of panel D) compared to simulations with increased recombination rate, mutation rate, age of the selected allele and reduced effective population size. Balancing selection was simulated under overdominance, and parameters in the reference set of simulations are N_e = 20,000, recombination and mutation rates of 10^{-8} per site per generation, selection onset time 160,000 generations ago. NCD target frequency was set to the true equilibrium frequency.

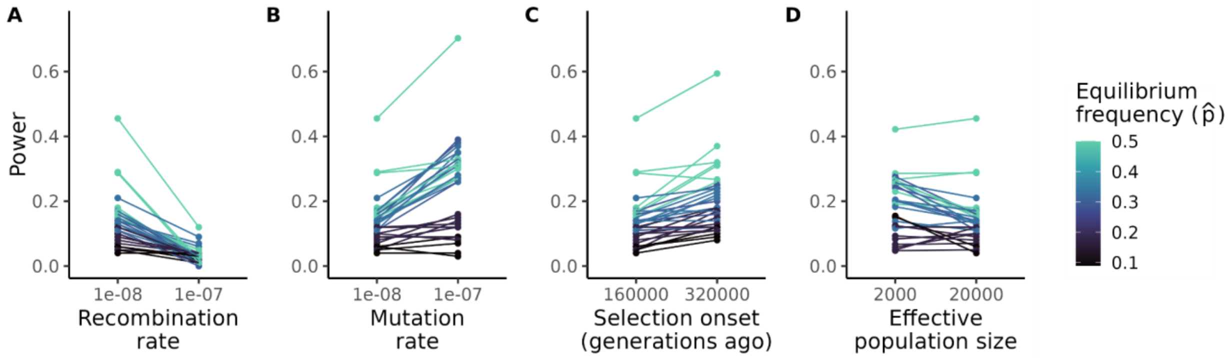

Table S9: Analysis of Deviance for GLM including equilibrium frequency (pstar), selection strength (selalpha), **recombination rate** (rec_recoded, as $-\log_{10}(r)$) and their interactions as predictor variables.

The table provides information from the Analysis of Deviance (degrees of freedom–Df, Deviance and their residuals, P-value, percentage of deviance explained–Perc Dev), the estimated slope (b) and the odds ratio (OR=exp(b)) for main effects. Predictors that include recombination rate, have a significant effect (p<0.05), and percentage of deviance explained higher than 1% are highlighted.

	Df	Deviance	Resid. Df	Resid. Dev	Pr(>Chi)	Perc Dev	b	OR
NULL	NA	NA	61	451.6	NA	NA	-9.52	0
pstar	1	88.0	60	363.7	0.000	19.5	-19.42	0
selalpha	1	27.5	59	336.2	0.000	6.1	1.99	7.326
**rec_recoded**	**1**	**249.9**	**58**	**86.3**	**0.000**	**55.3**	**-0.84**	**0.431**
pstar:selalpha	1	6.8	57	79.5	0.009	1.5	0.42	-
**pstar:rec_recoded**	**1**	**11.1**	**56**	**68.4**	**0.001**	**2.5**	**-2.77**	**-**
selalpha:rec_recoded	1	1.5	55	66.9	0.218	0.3	0.28	-
pstar:selalpha:rec_recoded	1	0.0	54	66.8	0.908	0.0	-0.15	-

Table S10: Analysis of Deviance for GLM including equilibrium frequency (pstar), selection strength (selalpha), **mutation rate** (mut_recoded, as -$\log_{10}(\mu)$) and their interactions as predictor variables.

The table provides information from the Analysis of Deviance (degrees of freedom–Df, Deviance and their residuals, P-value, percentage of deviance explained–Perc Dev), the estimated slope (b) and the odds ratio (OR=exp(b)) for main effects. Predictors that include mutation rate, have a significant effect (p<0.05), and percentage of deviance explained higher than 1% are highlighted.

	Df	Deviance	Resid. Df	Resid. Dev	Pr(>Chi)	Perc Dev	b	OR
NULL	NA	NA	61	595.4	NA	NA	4.2	68.598
pstar	1	280.4	60	315.0	0.000	47.1	-0.1	0.934
selalpha	1	33.5	59	281.4	0.000	5.6	-5.8	0.003
**mut_recoded**	**1**	**112.9**	**58**	**168.6**	**0.000**	**19.0**	**0.9**	**2.407**
pstar:selalpha	1	37.1	57	131.5	0.000	6.2	15.8	-
pstar:mut_recoded	1	1.4	56	130.1	0.239	0.2	-0.3	-
selalpha:mut_recoded	1	0.0	55	130.1	0.872	0.0	-0.7	-
pstar:selalpha:mut_recoded	1	4.0	54	126.1	0.046	0.7	1.8	-

Table S11: Analysis of Deviance for GLM including equilibrium frequency (pstar), selection strength (selalpha), **allelic age** (t_ne, in N_e generations) and their interactions as predictor variables.

The table provides information from the Analysis of Deviance (degrees of freedom–Df, Deviance and their residuals, P-value, percentage of deviance explained–Perc Dev), the estimated slope (b) and the odds ratio (OR=exp(b)) for main effects. Predictors that include allelic age, have a significant effect (p<0.05), and percentage of deviance explained higher than 1% are highlighted.

	Df	Deviance	Resid. Df	Resid. Dev	Pr(>Chi)	PercDev	b	OR
NULL	NA	NA	123	1087.3	NA	NA	-2.7	0.066
pstar	1	131.9	122	955.4	0.000	12.13	-1.9	0.154
selalpha	1	51.7	121	903.7	0.000	4.75	-0.2	0.832
**t_ne**	**1**	**557.1**	**120**	**346.6**	**0.000**	**51.24**	**0.0**	**1.034**
pstar:selalpha	1	35.2	119	311.4	0.000	3.24	2.3	-
**pstar:t_ne**	**1**	**69.2**	**118**	**242.2**	**0.000**	**6.36**	**0.3**	**-**
**selalpha:t_ne**	**1**	**12.5**	**117**	**229.7**	**0.000**	**1.15**	**0.0**	**-**
pstar:selalpha:t_ne	1	0.1	116	229.6	0.782	0.01	0.0	-

Table S6: Analysis of Deviance for GLM including equilibrium frequency (pstar), selection strength (selalpha), N_e (NeK, in thousands) and their interactions as predictor variables.

The table provides information from the Analysis of Deviance (degrees of freedom–Df, Deviance and their residuals, P-value, percentage of deviance explained–Perc Dev), the estimated slope (b) and the odds ratio (OR=exp(b)) for main effects. Predictors that include N_e, have a significant effect (p<0.05), and percentage of deviance explained higher than 1% are highlighted.

	Df	Deviance	Resid. Df	Resid. Dev	Pr(>Chi)	Perc Dev	b	OR
NULL	NA	NA	59	292.5	NA	NA	-2.822	0.059
pstar	1	163.3	58	129.2	0.000	55.8	3.592	36.32
selalpha	1	33.1	57	96.2	0.000	11.3	0.604	1.829
**NeK**	**1**	**15.4**	**56**	**80.7**	**0.000**	**5.3**	**0.001**	**1.001**
pstar:selalpha	1	1.9	55	78.8	0.167	0.7	-0.637	-
pstar:NeK	1	0.7	54	78.2	0.410	0.2	-0.044	-
selalpha:NeK	1	0.0	53	78.2	0.996	0.0	-0.043	-
**pstar:selalpha:NeK**	**1**	**4.4**	**52**	**73.8**	**0.036**	**1.5**	**0.113**	**-**

3. BalLeRMix optimizing equilibrium frequency

3.1. Effects of equilibrium frequency, selection strength and model of selection in reference simulations

Figure S19: Power of BalLeRMix as a function of equilibrium frequency ($\hat{p}$, panel A), selection strength (α, panel B), and model of selection (panel C).

Results shown are for reference simulations, and BalLeRMix was allowed to optimize the equilibrium frequency. On panels A and C points were horizontally jittered for visualization purposes. The three panels show the same data points, with power on the vertical axis plotted against different horizontal axes of variation.

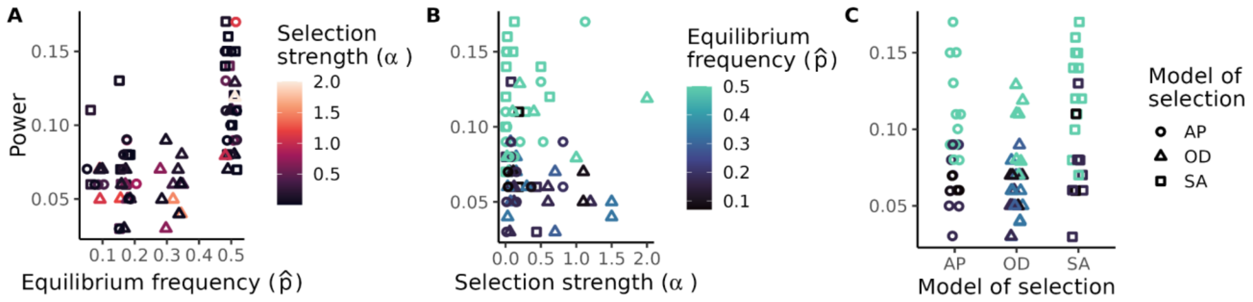

Table S13: Coefficients of a GLM for detection as a function of equilibrium frequency (pstar), strength of selection (selalpha), model of selection (seltype) and their interactions.

Significance codes: 0 '***' 0.001 '**' 0.01 '*' 0.05 '.' 0.1 ' ' 1.

	Estimate	Std. Error	z value	Pr(>|z|)	Significance
(Intercept)	-2.99287	0.2549	-11.741	<2e-16	***
pstar	1.27821	0.70762	1.806	0.0709	.
selalpha	-0.24576	0.35767	-0.687	0.492	
seltype_recodedAP	0.05709	0.3393	0.168	0.8664	
seltype_recodedSA	0.5409	0.35065	1.543	0.1229	
pstar:selalpha	0.60633	0.91524	0.662	0.5077	
pstar:seltype_recodedAP	0.26725	0.88963	0.3	0.7639	
pstar:seltype_recodedSA	-0.29893	0.9079	-0.329	0.742	
selalpha:seltype_recodedAP	0.29462	0.72275	0.408	0.6835	
selalpha:seltype_recodedSA	-1.60585	1.30088	-1.234	0.217	
pstar:selalpha:seltype_recodedAP	0.06676	1.72282	0.039	0.9691	
pstar:selalpha:seltype_recodedSA	3.29224	2.92498	1.126	0.2604	

**Table S14**: Analysis of Deviance for the model shown in Table S13. AIC: 367.26. Significance codes: 0 ‘***’ 0.001 ‘**’ 0.01 ‘*’ 0.05 ‘.’ 0.1 ‘ ’ 1.						
	Df	Deviance	Resid. Df	Resid. Dev	Pr(>Chi)	Significance
NULL			76	118.742		
pstar	1	49.769	75	68.973	1.73E-12	***
selalpha	1	1.046	74	67.928	0.306514	
seltype_recoded	2	13.429	72	54.499	0.001213	**
pstar:selalpha	1	1.285	71	53.214	0.256882	
pstar:seltype_recoded	2	0.331	69	52.883	0.847551	
selalpha:seltype_recoded	2	2.069	67	50.814	0.355424	
pstar:selalpha:seltype_recoded	2	1.323	65	49.491	0.516172	

3.2. Effects of recombination, mutation, selection onset and N_e in simulations of overdominance

Figure S20: Power of BalLeRMix as a function of recombination rate (panel A), mutation rate (panel B), selection onset time (panel C) and effective population size (panel D).

Power to detect balancing selection in our reference set of simulations (points on the left of panels A-C and on the right of panel D) compared to simulations with increased recombination rate, mutation rate, age of the selected allele and reduced effective population size. Balancing selection was simulated under overdominance, and parameters in the reference set of simulations are N_e = 20,000, recombination and mutation rates of 10^{-8} per site per generation, selection onset time 160,000 generations ago. BalLeRMix was allowed to optimize the equilibrium frequency.

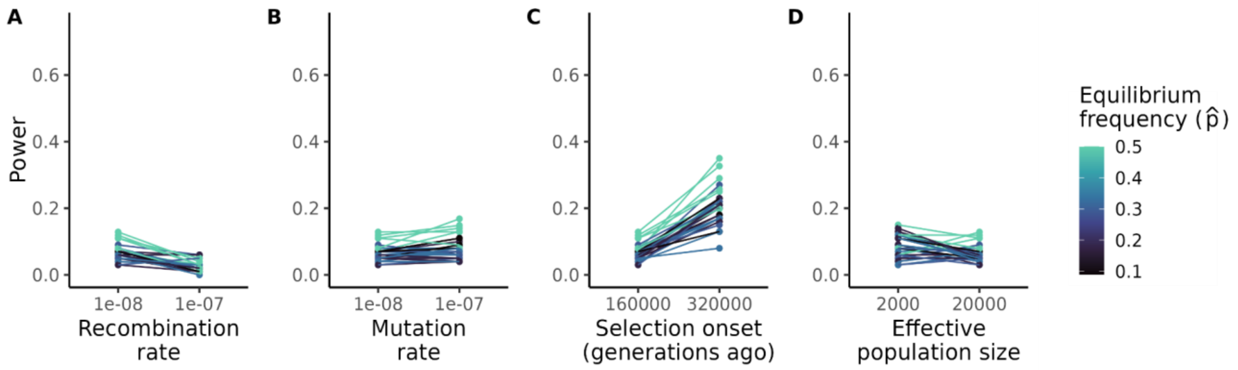

Table S15: Analysis of Deviance for GLM including equilibrium frequency (pstar), selection strength (selalpha), **recombination rate** (rec_recoded, as $-\log_{10}(r)$) and their interactions as predictor variables.

The table provides information from the Analysis of Deviance (degrees of freedom–Df, Deviance and their residuals, P-value, percentage of deviance explained–Perc Dev), the estimated slope (b) and the odds ratio (OR=exp(b)) for main effects. Predictors that include recombination rate, have a significant effect (p<0.05), and percentage of deviance explained higher than 1% are highlighted.

	Df	Deviance	Resid. Df	Resid. Dev	Pr(>Chi)	Perc. Dev	b	OR
NULL	NA	NA	61	118.6	NA	NA	-7.62	0.000
pstar	1	8.9	60	109.7	0.003	7.48	-8.68	0.000
selalpha	1	0.2	59	109.5	0.680	0.14	-1.84	0.159
**rec_recoded**	**1**	**61.7**	**58**	**47.8**	**0.000**	**52.06**	**-0.58**	**0.560**
pstar:selalpha	1	0.9	57	46.9	0.345	0.75	3.80	-
pstar:rec_recoded	1	1.2	56	45.7	0.274	1.01	-1.24	-
selalpha:rec_recoded	1	0.1	55	45.7	0.813	0.05	-0.20	-
pstar:selalpha:rec_recoded	1	0.1	54	45.6	0.815	0.05	0.40	-

Table S16: Analysis of Deviance for GLM including equilibrium frequency (pstar), selection strength (selalpha), **mutation rate** (mut_recoded, as -$\log_{10}(\mu)$) and their interactions as predictor variables.

The table provides information from the Analysis of Deviance (degrees of freedom–Df, Deviance and their residuals, P-value, percentage of deviance explained–Perc Dev), the estimated slope (b) and the odds ratio (OR=exp(b)) for main effects. Predictors that include mutation rate, have a significant effect (p<0.05), and percentage of deviance explained higher than 1% are highlighted.

	Df	Deviance	Resid. Df	Resid. Dev	Pr(>Chi)	Perc. Dev	b	OR
NULL	NA	NA	61	75.8	NA	NA	-0.3	0.723
pstar	1	21.6	60	54.2	0.000	28.51	-2.1	0.125
selalpha	1	0.0	59	54.2	0.998	0	-2.3	0.104
**mut_recoded**	**1**	**4.6**	**58**	**49.6**	**0.033**	**6.03**	**0.3**	**1.395**
pstar:selalpha	1	2.9	57	46.7	0.087	3.87	7.1	-
pstar:mut_recoded	1	0.0	56	46.7	0.969	0	-0.4	-
selalpha:mut_recoded	1	0.1	55	46.6	0.782	0.1	-0.3	-
pstar:selalpha:mut_recoded	1	0.4	54	46.2	0.508	0.58	0.8	-

Table S17: Analysis of Deviance for GLM including equilibrium frequency (pstar), selection strength (selalpha), **allelic age** (t_ne, in N_e generations) and their interactions as predictor variables.

The table provides information from the Analysis of Deviance (degrees of freedom–Df, Deviance and their residuals, P-value, percentage of deviance explained–Perc Dev), the estimated slope (b) and the odds ratio (OR=exp(b)) for main effects. Predictors that include allelic age, have a significant effect (p<0.05), and percentage of deviance explained higher than 1% are highlighted.

	Df	Deviance	Resid. Df	Resid. Dev	Pr(>Chi)	Perc. Dev	b	OR
NULL	NA	NA	123	1078.5	NA	NA	-4.69	0.009
pstar	1	16.6	122	1061.9	0.000	1.54	0.80	2.222
selalpha	1	2.9	121	1059.0	0.090	0.27	0.17	1.185
**t_ne**	**1**	**906.1**	**120**	**152.9**	**0.000**	**84.02**	**0.20**	**1.22**
pstar:selalpha	1	0.5	119	152.3	0.461	0.05	0.55	-
pstar:t_ne	1	0.0	118	152.3	0.971	0	0.02	-
selalpha:t_ne	1	9.6	117	142.7	0.002	0.89	-0.03	-
pstar:selalpha:t_ne	1	0.1	116	142.7	0.787	0.01	-0.02	-

Table S18: Analysis of Deviance for GLM including equilibrium frequency (pstar), selection strength (selalpha), N_e (NeK, in thousands) and their interactions as predictor variables.

The table provides information from the Analysis of Deviance (degrees of freedom–Df, Deviance and their residuals, P-value, percentage of deviance explained–Perc Dev), the estimated slope (b) and the odds ratio (OR=exp(b)) for main effects. Predictors that include N_e, have a significant effect (p<0.05), and percentage of deviance explained higher than 1% are highlighted.

	Df	Deviance	Resid. Df	Resid. Dev	Pr(>Chi)	Perc. Dev	b	OR
NULL	NA	NA	59	79.1	NA	NA	-2.80	0.061
pstar	1	5.6	58	73.5	0.018	7.06	0.64	1.894
selalpha	1	5.5	57	68.0	0.019	6.97	0.73	2.082
**NeK**	**1**	**6.8**	**56**	**61.2**	**0.009**	**8.59**	**-0.01**	**0.99**
pstar:selalpha	1	0.2	55	61.0	0.662	0.24	-0.91	-
**pstar:NeK**	**1**	**4.2**	**54**	**56.8**	**0.041**	**5.3**	**0.03**	-
**selalpha:NeK**	**1**	**5.2**	**53**	**51.6**	**0.022**	**6.6**	**-0.05**	-
pstar:selalpha:NeK	1	1.2	52	50.4	0.273	1.52	0.08	-

4. BalLeRMix setting equilibrium frequency to the theoretical value

4.1 Effects of equilibrium frequency, selection strength and model of selection in reference simulations

Figure S21: Power of BalLeRMix as a function of equilibrium frequency ($\hat{p}$, panel A), selection strength (α, panel B), and model of selection (panel C).

Results shown are for reference simulations, and BalLeRMix was provided the true equilibrium frequency. On panels A and C points were horizontally jittered for visualization purposes. The three panels show the same data points, with power on the vertical axis plotted against different horizontal axes of variation.

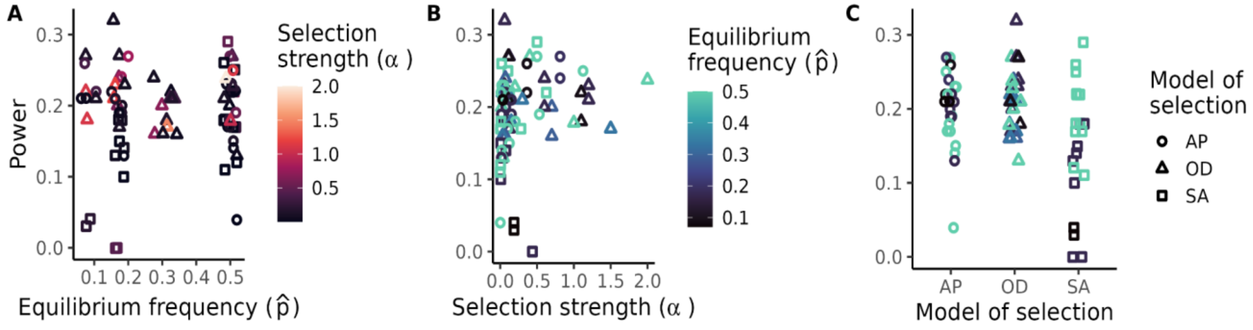

Table S19: Coefficients of a GLM for detection as a function of equilibrium frequency (pstar), strength of selection (selalpha), model of selection (seltype) and their interactions.

Significance codes: 0 '***' 0.001 '**' 0.01 '*' 0.05 '.' 0.1 ' ' 1.

	Estimate	Std. Error	z value	Pr(>|z|)	Significance
(Intercept)	-1.0293	0.1462	-7.038	1.95E-12	***
pstar	-0.8789	0.4398	-1.999	0.0457	*
selalpha	-0.3406	0.2016	-1.69	0.0911	.
seltype_recodedAP	-0.3405	0.2001	-1.702	0.0888	.
seltype_recodedSA	-0.4642	0.2613	-1.777	0.0756	.
pstar:selalpha	0.8652	0.5565	1.555	0.12	
pstar:seltype_recodedAP	0.4074	0.5712	0.713	0.4757	
pstar:seltype_recodedSA	0.8662	0.6699	1.293	0.196	
selalpha:seltype_recodedAP	0.8706	0.4334	2.009	0.0446	*
selalpha:seltype_recodedSA	-11.1898	2.1021	-5.323	1.02E-07	***
pstar:selalpha:seltype_recodedAP	-0.8783	1.1216	-0.783	0.4336	
pstar:selalpha:seltype_recodedSA	23.5921	4.328	5.451	5.01E-08	***

Table S20: Analysis of Deviance for the model shown in Table S19. AIC: 450.7.

Significance codes: 0 ‘***’ 0.001 ‘**’ 0.01 ‘*’ 0.05 ‘.’ 0.1 ‘ ’ 1.

	Df	Deviance	Resid. Df	Resid. Dev	Pr(>Chi)	Significance
NULL			76	243.043		
pstar	1	1.906	75	241.137	0.16739	
selalpha	1	4.814	74	236.323	0.028235	*
seltype_recoded	2	28.61	72	207.713	6.13E-07	***
pstar:selalpha	1	0.707	71	207.006	0.400446	
pstar:seltype_recoded	2	59.467	69	147.539	1.22E-13	***
selalpha:seltype_recoded	2	11.313	67	136.226	0.003495	**
pstar:selalpha:seltype_recoded	2	50.056	65	86.171	1.35E-11	***

4.2. Effects of recombination, mutation, selection onset and N_e in simulations of overdominance

Figure S22: Power of BalLeRMix as a function of recombination rate (panel A), mutation rate (panel B), selection onset time (panel C) and effective population size (panel D).

Power to detect balancing selection in our reference set of simulations (points on the left of panels A-C and on the right of panel D) compared to simulations with increased recombination rate, mutation rate, age of the selected allele and reduced effective population size. Balancing selection was simulated under overdominance, and parameters in the reference set of simulations are N_e = 20,000, recombination and mutation rates of 10^{-8} per site per generation, selection onset time 160,000 generations ago. BalLeRMix was provided with the true equilibrium frequency.

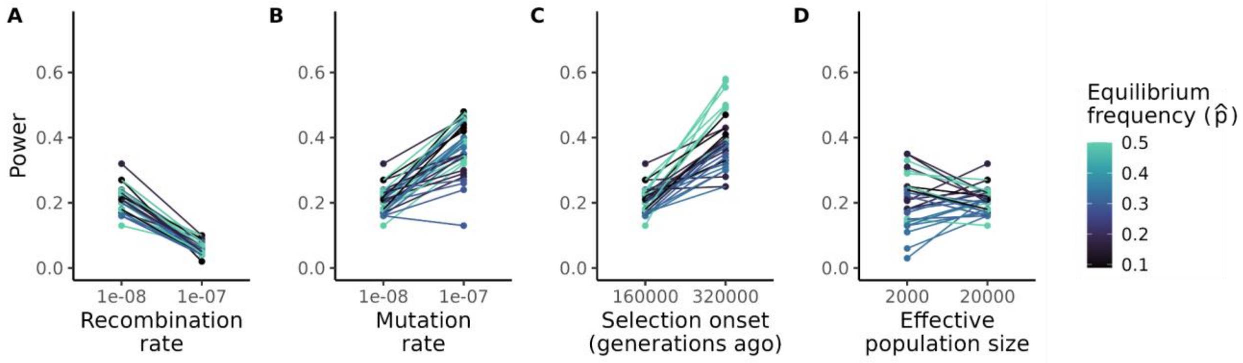

Table S21: Analysis of Deviance for GLM including equilibrium frequency (pstar), selection strength (selalpha), **recombination rate** (rec_recoded, as $-\log_{10}(r)$) and their interactions as predictor variables.

The table provides information from the Analysis of Deviance (degrees of freedom–Df, Deviance and their residuals, P-value, percentage of deviance explained–Perc Dev), the estimated slope (b) and the odds ratio (OR=exp(b)) for main effects. Predictors that include recombination rate, have a significant effect (p<0.05), and percentage of deviance explained higher than 1% are highlighted.

	Df	Deviance	Resid. Df	Resid. Dev	Pr (>Chi)	PercDev	b	OR
NULL	NA	NA	61	356.1	NA	NA	-13.39	0
pstar	1	1.3	60	354.8	0.263	0.35	4.23	69.048
selalpha	1	1.2	59	353.6	0.274	0.34	0.19	1.207
**rec_recoded**	**1**	**304.0**	**58**	**49.6**	**0.000**	**85.39**	**-1.54**	**0.213**
pstar:selalpha	1	2.3	57	47.3	0.128	0.65	-3.01	-
pstar:rec_recoded	1	0.4	56	46.9	0.542	0.1	0.64	-
selalpha:rec_recoded	1	0.3	55	46.6	0.563	0.09	0.07	-
pstar:selalpha:rec_recoded	1	0.2	54	46.4	0.664	0.05	-0.48	-

Table S22: Analysis of Deviance for GLM including equilibrium frequency (pstar), selection strength (selalpha), **mutation rate** (mut_recoded, as -$\log_{10}(\mu)$) and their interactions as predictor variables.

The table provides information from the Analysis of Deviance (degrees of freedom–Df, Deviance and their residuals, P-value, percentage of deviance explained–Perc Dev), the estimated slope (b) and the odds ratio (OR=exp(b)) for main effects. Predictors that include mutation rate, have a significant effect (p<0.05), and percentage of deviance explained higher than 1% are highlighted.

	Df	Deviance	Resid. Df	Resid. Dev	Pr (>Chi)	PercDev	b	OR
NULL	NA	NA	61	296.7	NA	NA	3.55	34.673
pstar	1	0.6	60	296.1	0.452	0.19	3.38	29.511
selalpha	1	0.0	59	296.1	0.876	0.01	0.78	2.171
**mut_recoded**	**1**	**181.8**	**58**	**114.3**	**0.000**	**61.29**	**0.57**	**1.772**
pstar:selalpha	1	4.4	57	109.8	0.035	1.49	-0.62	-
pstar:mut_recoded	1	1.1	56	108.7	0.293	0.37	0.53	-
selalpha:mut_recoded	1	0.5	55	108.2	0.466	0.18	0.14	-
pstar:selalpha:mut_recoded	1	0.1	54	108.1	0.798	0.02	-0.19	-

Table S23: Analysis of Deviance for GLM including equilibrium frequency (pstar), selection strength (selalpha), **allelic age** (t_ne, in N_e generations) and their interactions as predictor variables.

The table provides information from the Analysis of Deviance (degrees of freedom–Df, Deviance and their residuals, P-value, percentage of deviance explained–Perc Dev), the estimated slope (b) and the odds ratio (OR=exp(b)) for main effects. Predictors that include allelic age, have a significant effect (p<0.05), and percentage of deviance explained higher than 1% are highlighted.

	Df	Deviance	Resid. Df	Resid. Dev	Pr(>Chi)	PercDev	b	OR
NULL	NA	NA	61	373.8	NA	NA	-1.37	0.253
pstar	1	6.9	60	366.9	0.009	1.84	-2.68	0.069
selalpha	1	0.5	59	366.4	0.464	0.14	-0.48	0.621
**t_ne**	**1**	**247.1**	**58**	**119.2**	**0.000**	**66.12**	**0.04**	**1.044**
pstar:selalpha	1	3.4	57	115.8	0.065	0.91	1.20	-
**pstar:t_ne**	**1**	**15.3**	**56**	**100.5**	**0.000**	**4.1**	**0.23**	**-**
selalpha:t_ne	1	0.0	55	100.4	0.836	0.01	0.02	-
pstar:selalpha:t_ne	1	0.2	54	100.2	0.632	0.06	-0.04	-

Table S24: Analysis of Deviance for GLM including equilibrium frequency (pstar), selection strength (selalpha), N_e (NeK, in thousands) and their interactions as predictor variables.

The table provides information from the Analysis of Deviance (degrees of freedom–Df, Deviance and their residuals, P-value, percentage of deviance explained–Perc Dev), the estimated slope (b) and the odds ratio (OR=exp(b)) for main effects. Predictors that include N_e, have a significant effect (p<0.05), and percentage of deviance explained higher than 1% are highlighted.

	Df	Deviance	Resid. Df	Resid. Dev	Pr(>Chi)	PercDev	b	OR
NULL	NA	NA	58	150.4	NA	NA	-1.64	0.194
pstar	1	4.1	57	146.3	0.044	2.69	-0.05	0.951
selalpha	1	10.4	56	135.9	0.001	6.92	0.70	2.022
NeK	1	0.1	55	135.8	0.710	0.09	0.03	1.031
pstar:selalpha	1	0.6	54	135.2	0.446	0.39	-0.62	-
pstar:NeK	1	0.1	53	135.1	0.751	0.07	-0.04	-
**selalpha:NeK**	**1**	**18.0**	**52**	**117.1**	**0.000**	**11.99**	**-0.05**	**-**
pstar:selalpha:NeK	1	2.6	51	114.4	0.105	1.75	0.07	-
